# Non-viral delivery of genome-editing tools for treatment of genetic disorders

**DOI:** 10.1016/j.apsb.2026.02.014

**Published:** 2026-02-23

**Authors:** Jiamin Yang, Yuxuan Chen, Xiaohong Chen, Yuan Ping

**Affiliations:** a College of Pharmaceutical Sciences, Zhejiang University, Hangzhou 310058, China; b Liangzhu Laboratory, Zhejiang University Medical Center, Hangzhou 311121, China; c Zhejiang Provincial Key Laboratory of Pancreatic Disease, MOE Joint International Research Laboratory of Pancreatic Diseases, The First Affiliated Hospital, Zhejiang University School of Medicine, Hangzhou 310003, China

**Keywords:** Genetic diseases, Genome editing, Gene therapy, CRISPR-Cas, Non-viral delivery, Lipid nanoparticle, Virus-like particle, Selective organ targeting

## Abstract

Pathogenic mutations within protein-coding regions of genomic DNA can disrupt protein structure and lead to hereditary disorders. Genome-editing technologies, particularly those based on clustered, regularly interspaced, short palindromic repeats-associated protein (CRISPR-Cas), are promising therapeutic tools for correcting genetic abnormalities. To date, viral delivery vectors for genome-editing biomacromolecules have shown numerous promises in treating genetic disorders. However, safe viral delivery for genome-editing components remains challenging, largely due to the immunogenicity of viruses. As an alternative, non-viral delivery systems are emerging as a safer choice and may offer solutions to address the safety challenges. In this review, we first introduce CRISPR-Cas9-based genome editing tools and their delivery formats. Then, we outline the pathology of major genetic disorders and both preclinical and clinical approaches for these diseases by therapeutic genome editing, and provide an overview of current non-viral delivery strategies and their potential to overcome existing limitations. Finally, we discuss the current challenges and future outlooks of non-viral delivery of gene-editing components in treating genetic diseases.

## Graphical abstract

Gene-editing systems can be delivered by five principal classes of non-viral vectors for the treatment of genetic diseases. Through rational engineering of delivery vehicles, gene-editing cargos can be directed to specific tissues, enabling precise therapy for diverse diseases.

**Figure:**
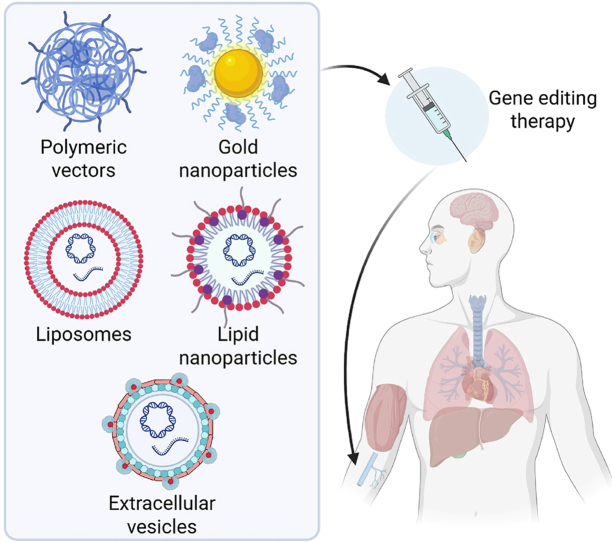

## 1 Introduction

Mutations in the coding regions of genomic DNA can cause protein malfunction and constitute the basis of various genetic disorders (Table 1). Rare diseases affect an estimated 300 million people globally and are a major contributor to child mortality and disability, particularly in high-income countries^1^. A recent BabyDetect pilot project found that about 1.8% of newborns (71 out of 3847) had severe and rare genetic conditions, including 44 cases of glucose-6-phosphate dehydrogenase deficiency, 5 cases of cystic fibrosis (CF), 1 case of phenylketonuria (PKU), among others^2^. Despite decades of efforts, effective treatments that correct DNA-level mutations remain rare^3^. Recent advances in genome-editing technologies offer the potential to permanently correct genetic mutations, offering new treatment opportunities. Unlike conventional gene therapies that rely on transgene expression or RNA interference^3^, ^4^, ^5^, gene-editing technologies directly correct pathogenic mutations in the somatic cells. Several *ex vivo* and *in vivo* genome-editing therapeutics are currently in clinical trials for a broader spectrum of genetic diseases, including hereditary tyrosinemia type 1 (HT1) and transthyretin amyloidosis (ATTR) (Table 2). Three major categories of programmed nucleases—zinc-finger nucleases (ZFNs), transcription activator-like effector nucleases (TALENs), and clustered, regularly interspaced, short palindromic repeats (CRISPR) associated proteins (CRISPR-Cas)—have been extensively used to treat genetic disorders in various animal models^6^^,^^7^. More recently, new gene-editing tools such as base editors (BEs) and prime editors (PEs) have opened new avenues for robust gene editing in mammalian cells^8^, ^9^, ^10^.

**Table 1:** An overview of genetic diseases introduced in this review.

Disease	Mutated gene	Pathogenic cause	Onset organ
HT1	*FAH*	Accumulation of maleylacetoacetic acid and fumarylacetoacetic acid	Liver
FH	*LDLR*, *APOB*, *PCSK9*	High levels of LDL-C in the blood	Liver
ATTR	*TTR*	Misfolding and aggregation of TTR	Heart, liver
HAE	*SERPING1*	Insufficient C1INH activity	Liver, skin, gastrointestinal tract
PKU	*PAH*	Deficiency of the enzyme phenylalanine hydroxylase	Liver
SP-B deficiency	*SFTPB*	SP-B deficiency	Lung
CF	*CFTR*	Defective or absent CFTR protein	Lung, pancreas
Mucopolysaccharidoses	*IDUA*, *IDS*	Deficiency of enzymes required for GAGs degradation within lysosomes	Skeletal system, central nervous system
DMD	*DMD*	Deficiency of the dystrophin protein	Skeletal muscle
LCA10	*CEP290*	Misfunction of photoreceptor cells	Retina
HL	*TMC1*	Dysfunction of mechanically activated channels in hair cells	Ear
FXS	*FMR1*	The loss of FMRP	Brain
SCD	*HBB*	Production of abnormal hemoglobin S	Blood

Abbreviations: ATTR, transthyretin amyloidosis; C1INH, C1 inhibitor; CF, cystic fibrosis; CFTR, cystic fibrosis transmembrane conductance regulator; DMD, Duchenne muscular dystrophy; FH, familial hypercholesterolemia; FMRP, fragile X mental retardation protein; FXS, fragile X syndrome; GAGs, glycosaminoglycans; HAE, hereditary angioedema; HL, hearing loss; HT1, hereditary tyrosinemia type 1; LCA10, Leber congenital amaurosis type 10; LDL-C, low-density-lipoprotein cholesterol; PKU, phenylketonuria; SCD, sickle cell disease; SP-B, surfactant protein B; TTR, transthyretin.

**Table 2:** Gene editing therapy in clinical trials for treating genetic disease.

Therapy	Disease	ClinicalTrials.gov identifier	Status	Vectors	*In/ex vivo*
VERVE-101	Heterozygous FH	NCT05398029	Active, not recruiting	LNP	*In vivo*
NTLA-2001	ATTR amyloidosis	NCT06128629	Recruiting	LNP	*In vivo*
NTLA-2002	HAE	NCT05120830	Active, not recruiting	LNP	*In vivo*
SB-318	MPS I	NCT05514249	Terminated	AAV2/6	*In vivo*
SB-913	MPS II	NCT03041324	Terminated	AAV2/6	*In vivo*
CRD-TMH-001	DMD	NCT05514249	Active, not recruiting	AAV9	*In vivo*
EDIT-101	LCA10	NCT03872479	Active, not recruiting	AAV5	*In vivo*
SB-FIX	Hemophilia B	NCT02695160	Terminated	AAV2/6	*In vivo*
CTX001	Transfusion-dependent *β*-thalassemia	NCT03655678	Active, not recruiting	Electroporation	*Ex vivo*
	SCD	NCT05329649 NCT05477563	Recruiting		*Ex vivo*
ET-01	Transfusion dependent *β*-thalassemia	NCT04925206	Active, not recruiting	Electroporation	*Ex vivo*
BEAM-101	SCD	NCT05456880	Recruiting	Electroporation	*Ex vivo*
	*β*-thalassemia	NCT05456880	Recruiting		*Ex vivo*
EDIT-301	Transfusion-dependent *β*-thalassemia	NCT05444894	Recruiting	Electroporation	*Ex vivo*
ZVS203e	Retinitis pigmentosa	NCT05805007	Recruiting	rAAV	*Ex vivo*
BRL-101	Transfusion-dependent *β*-thalassemia	NCT06298630	Not yet	Electroporation	*Ex vivo*

Abbreviations: AAV, adeno-associated virus; ATTR, transthyretin amyloidosis; DMD, Duchenne muscular dystrophy; FH, familial hypercholesterolaemia; HAE, hereditary angio-oedema; LCA10, Leber congenital amaurosis type 10; LNP, lipid nanoparticle; MPS I, mucopolysaccharidosis type I; MPS II, mucopolysaccharidosis type II; rAAV, recombinant adeno-associated virus; SCD, sickle cell disease.

Effective treatment of genetic diseases requires the precise delivery of gene-editing components into mutated cells. To accomplish this, various delivery vectors have been designed and evaluated in mice, non-human primates (NHPs), and human clinical trials^6^^,^^7^^,^^11^^,^^12^. These vectors generally fall into two categories: viral and non-viral. Viral vectors, especially adeno-associated vectors (AAVs), have been widely utilized to deliver genome-editing components. Clinical trials have shown initial success in treating mucopolysaccharidosis (MPS) Type I (MPS I)^13^^,^^14^, mucopolysaccharidosis Type II (MPS II)^15^, and Leber congenital amaurosis type 10 (LCA10)^16^. Despite these advances, viral vectors bear inherent drawbacks, including immunogenicity, limited packaging capacity, and the risk of detrimental transgene insertion^17^. For example, Jesse Gelsinger’s death from systemic inflammatory response syndrome in 1999 led to the withdrawal of adenoviral vectors from frontline use^18^. And in 2002, a patient with X-linked severe combined immunodeficiency developed leukemia following retroviral gene therapy, demonstrating the risk of insertional oncogenesis^19^. In contrast, non-viral vectors offer advantages such as the ability to load different formats of gene-editing components, improved immune biocompatibility, and greater packing capacity^17^^,^^20^^,^^21^. These merits indicate the potential of non-viral delivery vectors for therapeutic genome editing.

**Figure 1:**
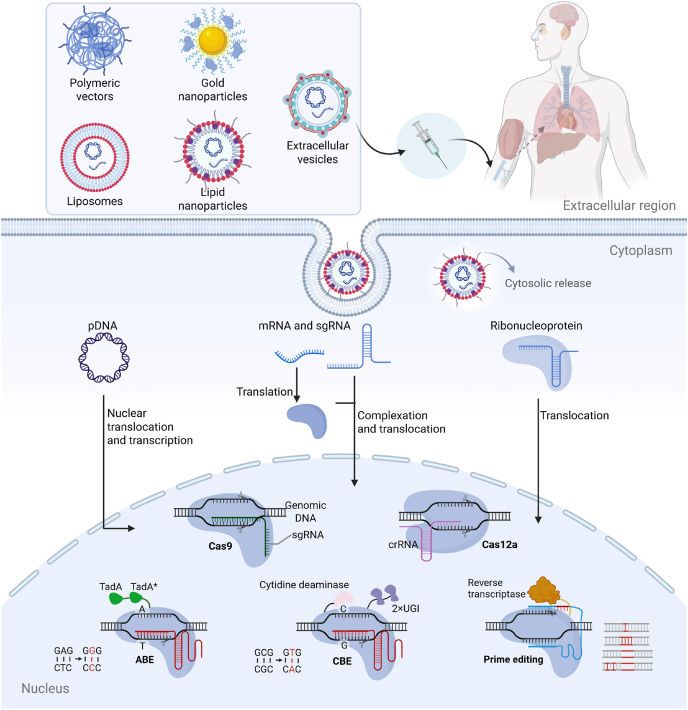
Delivery of therapeutic gene editing machinery using non-viral vectors for treating genetic diseases. Five representative carriers—lipid nanoparticles, liposomes, polymeric vectors, gold nanoparticles, and extracellular vesicles—deliver three cargo formats (plasmid DNA encoding Cas9 and sgRNA; Cas9 mRNA + sgRNA; or a Cas9–sgRNA ribonucleoprotein complex) into the cytoplasm, from which the cargos translocate to the nucleus. There, genome editors—including CRISPR-Cas9, Cas12a, adenine and cytosine base editors, and prime editors—execute sequence-specific corrections.

In this review, we first provide an overview of genome-editing tools that have been used in treating genetic diseases and their delivery formats, including plasmid DNA (pDNA), messenger RNA (mRNA), and ribonucleoprotein (RNP) complexes (Fig. 1). We then introduce several CRISPR-based gene-editing tools, including CRISPR-Cas9, CRISPR-Cas12a, BEs, PEs, and RNA editing tools. Then, we elucidate the pathophysiology of various genetic disorders and describe both preclinical and clinical genome-editing approaches. Given the crucial role of delivery in effective gene therapy, we highlight recent advances in non-viral delivery vectors, including gold nanoparticles (GNPs), polymeric vectors, lipid-based vectors, and virus-like particles (VLPs), for *in vivo* therapies. Finally, we summarize the progress of non-viral vectors and discuss challenges faced when translating these gene-editing therapies from bench to bedside.

## 2 Genome editors

Genome editing has significantly expanded its toolbox in recent years. Notable examples, such as ZFNs, TALENs, CRISPR-Cas9, CRISPR-Cas12a, BEs, PEs, and RNA editing tools, have all been employed to treat genetic diseases (Fig. 1). These editors can be delivered in various cargo formats. For example, the CRISPR-Cas9 system, comprising the Cas9 protein and single guide RNA (sgRNA), can be delivered in three formats: (1) as DNA encoding Cas9 protein and guide RNA (gRNA), (2) as mRNA that facilitates Cas9 translation—often co-delivered with sgRNA, and (3) as RNP complexes combining Cas9 protein with sgRNA^17^. Each delivery strategy features distinct characteristics, with unique advantages and challenges^22^.

### 2.1 Introduction of genome editors

#### 2.1.1 ZFNs and TALENs

ZFNs facilitate gene editing by fusing a recognition domain of zinc finger protein (ZFP) with the cleavage domain of the FokI restriction enzyme^23^. ZFPs consist of multiple zinc finger domains, each stabilized by binding a zinc ion through conserved cysteine and histidine residues and recognizing about three base pairs of DNA^24^. Conversely, the FokI domain cleaves DNA nonspecifically and requires dimerization for its activity^25^. By modifying both the zinc fingers and the FokI dimerization, ZFNs have been optimized to enhance gene editing specificity while reducing toxicity levels^26^. Similarly, TALENs combine a DNA-binding domain derived from transcription activator-like effector (TALE) with the FokI catalytic domain^27^. TALENs achieve DNA specificity through a series of 33–35 amino acid repeats, each containing a pair of hypervariable residues that recognize a single base pair^28^^,^^29^. This one-to-one recognition mechanism simplifies the modular design of TALENs compared to ZFNs^30^.

#### 2.1.2 CRISPR-Cas9

CRISPR-Cas is a family of programmable nucleases initially discovered for their ability to induce site-specific double-strand DNA breaks (DSBs), which has greatly advanced genome-editing technologies. The widely used archetypal Cas9 protein from *Streptococcus pyogenes* (spCas9), part of the type II CRISPR-Cas system, is preferred for its high specificity and efficacy^31^. The CRISPR-Cas9 system comprises three essential components: the Cas9 enzyme, responsible for DNA catalytic activity; CRISPR RNA (crRNA), serving as the guiding sequence; and *trans*-activating crRNA (tracrRNA), complementing crRNA and facilitating its maturation^31^^,^^32^. A simplified approach utilizes a single guide RNA (sgRNA) that is formed by fusing crRNA and tracrRNA to direct CRISPR-Cas9 to its target sites^31^^,^^33^. Target binding requires recognition of a short protospacer adjacent motif (PAM) downstream of the target site. Upon PAM recognition, the Cas9 protein unwinds the adjacent DNA and checks for complementarity with the sgRNA. If the match is sufficient, Cas9 undergoes conformational changes that activate its nuclease domains^34^. Cas9 then cleaves the double-stranded DNA (dsDNA) at specific locations to generate DSBs. These breaks are repaired through two distinct pathways: error-prone nonhomologous end joining (NHEJ), which typically results in small insertions or deletions (indels) that disrupt the gene, and homology-directed repair (HDR), which allows gene repair or insertion *via* template-guided mechanisms^35^.

#### 2.1.3 CRIPSR-Cas12a

Cas12a, also known as Cpf1, is a protein identified in Class 2, type V CRISPR-Cas systems^36^. In contrast to the CRISPR-Cas9 system, Cas12a processes its guides by recognizing a conserved pseudoknot structure within the repeat-derived segment of the crRNA^37^. Moreover, Cas12a recognizes a T-rich PAM, which distinguishes it from the G-rich PAM recognized by the CRISPR-Cas9 system^36^. Cas12a cleaves both DNA strands at sites to the PAM, generating DSBs with staggered ends. Additionally, after cleaving the target DNA, Cas12a remains catalytically active and can nonspecifically cleave additional single-stranded DNA (ssDNA), a property that has been harnessed for molecular diagnostic tools^38^.

#### 2.1.4 BEs

BEs are innovative genome-editing tools that enable precise, single-nucleotide modifications in DNA or RNA without inducing DSBs^39^. These tools function by combining a catalytically impaired Cas protein with a deaminase enzyme, which facilitates the conversion of one base to another at a specific genomic site^39^. There are two primary types of DNA BEs: cytosine base editors (CBEs) and adenine base editors (ABEs). CBEs convert C•G base pairs into T•A base pairs, while ABEs change A•T base pairs into G•C base pairs^39^. CBEs are engineered by fusing cytidine deaminases and uracil glycosylase inhibitor (UGI) domains with Cas9 nickase (nCas9)^40^. nCas9 specifically cleaves a single DNA strand to create a single-stranded stretch of genomic DNA (the R-loop). Then, cytidine deaminases facilitate the conversion of cytosines to uracils within the R-loop, which is read as a thymine during DNA replication, DNA repair, and transcription. Additionally, UGIs prevent the formation of abasic sites by inhibiting the activity of uracil glycosylases. Because natural deaminases that act on deoxyadenosine are not known, ABEs utilize laboratory-evolved TadA∗ deoxyadenosine deaminases to convert adenosines into inosines, which are then read as guanines during transcription^41^,^42^.

BEs are valuable for genome editing due to: (i) their high efficiency in non-dividing cells; (ii) the ability to bypass HDR machinery; (iii) the provision of precise editing outcomes with minimal byproducts (a >10:1 ratio of desired edits to indels); and (iv) their capability to function solely with mRNA or RNP agents, eliminating the need for DNA templates. Nevertheless, BEs are limited by the need for precise targeting within the optimal editing window, typically nucleotides 4–7 of the protospacer, to avoid unintended edits. Meanwhile, proper alignment with the PAM sequence is crucial for Cas9 binding; if the protospacer is misaligned, efficient editing may be hindered. Meanwhile, off-target editing remains a concern for BEs. To address these limitations, advanced versions, such as BE3, BE4, BE4-Gam, ABE7.10, and ABE8e^40^^,^^43^, ^44^, ^45^, have been developed to enhance editing efficiency and reduce indel occurrences. Furthermore, Walton et al.^46^ have developed SpG and SpRY variants of the spCas9 enzyme that can recognize a wider range of PAMs, overcoming the limitation faced by BEs in targeting only specific sequences within a genome.

To conclude, both ABEs^47^, ^48^, ^49^ and CBEs^50^ have demonstrated promising results in preclinical studies targeting genetic diseases. Considering that single-nucleotide polymorphisms cause many genetic diseases, base editing holds great promise for correcting such pathogenic mutations^51^^,^^52^.

#### 2.1.5 PEs

Conventional BEs are typically limited to correcting only four transition mutations (C→T, G→A, A→G, and T→C), In contrast, the prime editor developed by David Liu’s group^53^ can correct all 12 possible point mutations, as well as perform insertions up to 44 bp and deletions up to 80 bp, without inducing DSBs or necessitating donor DNA. The prime editing system comprises a fusion protein consisting of nCas9 (H840A) and reverse transcriptase (RT), along with a specifically designed guide RNA known as a prime editing guide RNA (pegRNA). Unlike standard sgRNA, pegRNA i contains an extra 3′ sequence that acts as a reverse RNA template and a primer binding site capable of hybridizing with the 3′ end of the nicked DNA strand. Upon target binding, the prime editor (PE2) creates a nick in the non-PAM-containing DNA strand at the R-loop. Reverse transcriptase then extends the 3′ end of the nicked strand using the pegRNA as a template, incorporating the desired edit into the newly synthesized DNA strand. The cellular DNA repair then processes the edited DNA flap, leading to its permanent integration into the genome.

PEs are valuable for genome editing due to their (i) high versatility compared to BEs while avoiding DSBs, (ii) cleaner editing outcomes with fewer indels than nuclease-mediated HDR, and (iii) exceptional precision with minimal off-target effects. This precision likely results from two extra DNA hybridization steps (reverse-transcriptase priming and DNA flap resolution) that help reject off-target sequences. However, while offering versatility, the editing efficiencies of PEs are limited, typically ranging from 5% to 20%, depending on the specific conditions. Moreover, the design of pegRNAs remains challenging. PEs require careful consideration of the editing window, template sequences, and the 3′ extension.

To enhance editing efficiency, PE3 introduces an additional nick on the opposite (non-edited) strand, which stimulates cellular repair mechanisms that favor the incorporation of the edited sequence^42^^,^^53^. Furthermore, PE4 and PE5, derived from PE2 and PE3, respectively, further improve editing efficiency by transiently co-expressing an engineered DNA mismatch repair (MMR)-inhibiting protein (MLH1dn)^54^. Additionally, PE6, developed through phage-assisted continuous evolution (PACE) and protein engineering, enhances the RT domain and Cas9 variants within the prime editing system, thereby improving its editing capabilities^55^. Recently, a novel system, PE7, has utilized the La RNA-binding N-terminal domain for specific recognition and interaction with the 3′ ends of polyuridylated pegRNAs, further improving the precision and efficiency of PEs^56^. As a versatile and precise tool, prime editing has significant potential in the treatment of genetic diseases.

#### 2.1.6 RNA editing

In addition to the DNA-editing tools mentioned above, some systems have also been developed for RNA editing^57^. For example, Cas13 utilizes crRNA for RNA recognition, with enzymatic versatility that includes precursor crRNA processing, sequence-specific RNA cleavage, and collateral RNA degradation, making it a promising candidate for RNA-based intervention^58^. For instance, Cas13a enables transient suppression of endogenous gene expression in human cell lines with high specificity and minimal off-target activity^59^. Recent advancements include engineered variants such as RfxCas13d and the compact Cas13X, which mitigate collateral RNA cleavage while retaining targeting precision^60^^,^^61^. Furthermore, catalytically inactive dCas13 fused with RNA-modifying deaminases has facilitated the development of RNA BEs, enabling precise correction of single-nucleotide mutations without permanent genomic alteration^62^^,^^63^. In the meantime, critical challenges, including bystander RNA effects, potential DNA off-target activity (despite RNA specificity), and immunogenicity, necessitate rigorous safety evaluations in preclinical models.

#### 2.1.7 Comparison of different genome editors

In summary, different genome editors have distinct features and applications. ZFNs and TALENs can recognize and bind to different DNA sequences, but the construction process is complex and costly. In contrast, CRISPR-Cas9 offers greater simplicity, efficiency, and versatility, making it the most widely adopted genome-editing tool for treating genetic diseases. However, compared to ZFNs and TALENs, which can target any target site, the CRISPR-Cas system is often limited by the PAM sequence. Additionally, CRISPR-Cas still carries the risk of off-target effects. In contrast, BEs and PEs directly convert single bases without inducing DSBs, thereby reducing potential safety concerns. Compared to BEs, PEs offer higher versatility, correcting a broader range of point mutations, offering higher accuracy, and lower off-target rates. However, BEs and PEs, due to their larger size and more complex components, pose new challenges for delivery. Compared to the aforementioned DNA-editing tools, RNA editing tools such as CRISPR-Cas13 can correct mutations at the transcriptional level, making them relatively safer. Nevertheless, because RNA molecules have a transient lifespan, continuous delivery of these editing tools may be required to achieve long-term therapeutic effects. Moreover, as the therapeutic potential of CRISPR technologies continues to grow, other important considerations, such as affordability, regulation, and access, must also be addressed. For a more comprehensive exploration of the ethical and societal challenges associated with these technologies, we direct readers to the relevant references^64^^,^^65^.

### 2.2 Delivery forms of gene editing cargoes

#### 2.2.1 Plasmid DNA

The first delivery format is plasmid DNA (pDNA), which encodes Cas9 and sgRNA, requiring nuclear entry in the host for transcription and translation into the Cas9 protein. Consequently, DNA exhibits slower expression kinetics compared to RNA and RNP complexes. Despite this, pDNA remains popular due to its straightforward production and stable expression characteristics^66^. An advantage of pDNA lies in its facile and convenient bacterial amplification, allowing for easy production. Moreover, DNA offers greater stability with the potential for either integration into the host chromosome or retention as episomal DNA within the nucleus. This long-term overexpression is preferred for some treatments, such as gene replacement therapies targeting recessive monogenic diseases^67^, ^68^, ^69^. However, utilizing DNA-based delivery formats may result in unintended genomic integration and potentially induce insertional mutagenesis^22^^,^^70^, and pDNA derived from bacteria might trigger immune responses *via* Toll-like receptors^71^. These limitations raise safety concerns when considering the transition of pDNA-based genome-editing tools from bench to bedside.

#### 2.2.2 RNA

For the RNA-based strategy, the co-delivery of two individual components—Cas9 mRNA and sgRNA—is used in the CRISPR-Cas system. RNA delivery offers distinct advantages over DNA-based delivery methods by addressing several inherent limitations. Specifically, Cas9 delivered as mRNA only requires cytoplasmic entry for translation, which provides two significant benefits: it circumvents obstacles related to nuclear localization of nucleic acids and reduces the risk of chromosomal integration associated with pDNA delivery. Furthermore, the naturally short half-life of mRNA facilitates rapid and transient Cas9 expression, minimizing off-target effects^72^. However, delivering mRNA and gRNA systems introduces new challenges. For example, *in vivo* kinetics differ between mRNA and sgRNA, necessitating an optimal co-delivery strategy that involves separate, staged delivery. Both components ultimately enter the same cell to form RNPs and achieve their intended function, which presents a paradoxical situation^73^. Moreover, suitable protection during *in vivo* delivery is essential due to the inherent instability and susceptibility to degradation exhibited by mRNA^72^.

Optimizing the structural design of mRNA systematically can boost protein expression, thereby increasing its efficacy in the treatment of genetic disorders^74^^,^^75^. For instance, the 5′ untranslated region (5′-UTR) and 3′ untranslated region (3′-UTR) exert influence on mRNA translation efficiency by modulating both mRNA stability and the translation process^76^. Additionally, the incorporation of alternative nucleotides, such as pseudouridine, *N*^6^-methyladenosine, and 5-methylcytidine, into mRNA can reduce immunogenicity and enhance translation efficiency^77^. Hence, mRNA delivery remains a promising field within gene-editing therapies.

#### 2.2.3 RNP

Cas9/gRNA RNPs are formed by combining the Cas9 protein with gRNA prior to introducing the complex into a cell. Upon binding to gRNA, the *α*-helix recognition lobe of the Cas9 protein undergoes a conformational change, thereby activating the protein for DNA recognition and cleavage^78^. As pre-assembled and functional RNP complexes, they enable the fastest kinetic form of genome editing^79^. The utilization of Cas9 RNPs offers several advantages over pDNA and RNA delivery approaches. RNPs can achieve higher gene editing efficiency with lower toxicity, resulting in reported on-target mutation rates ranging from 76% to 79%^70^. Furthermore, RNPs exhibit rapid degradation kinetics; *in vitro* studies have shown complete degradation of Cas9 proteins within 4 h, significantly reducing the risk of off-target effects^70^^,^^80^. Moreover, RNPs exhibit relatively low immunogenicity and may provide improved safety profiles for *in vivo* applications^81^. Despite the evident potential, the delivery of RNPs presents significant challenges for non-viral carriers due to the considerable size and complex charged residues of Cas9, as well as the strong negative charge of sgRNAs. Additionally, there is a need to protect RNPs from degradation or denaturation throughout the formulation and delivery process^82^. Nevertheless, continuous advancements in protein delivery research make it possible to overcome these challenges in the future.

#### 2.2.4 Comparison of pDNA, RNA, and RNP in non-viral delivery systems

In non-viral delivery systems, each format—pDNA, RNA, and RNP—has its unique advantages and limitations. pDNA remains popular for long-term gene expression due to its stability, ease of production, and potential for sustained overexpression. However, it carries the risk of genomic integration, potential immune activation, and slower expression kinetics. RNA and RNP complexes, on the other hand, offer a faster and safer alternative by bypassing genomic integration and reducing immune response. Their transient nature and instability, however, limit their application in therapies requiring long-term expression. Meanwhile, RNP complexes still face challenges in delivery efficiency and complex manufacturing processes, which can hinder their use in certain applications.

Notably, none of these forms can naturally pass through cell membranes, making the development of delivery strategies a critical challenge. While nucleic acids (pDNA and RNA) share similar chemical and physicochemical properties, proteins are highly diverse biomolecules with varying structural conformations, charges, and hydrophilicity/hydrophobicity. Therefore, the choice of delivery format must be carefully considered, taking into account the specific therapeutic goals, the required duration of expression, and safety considerations for each application.

## 3 Genome editing gene therapy for genetic diseases

Genome editors, particularly CRISPR-based technologies, have shown tremendous promise in preclinical and clinical studies for correcting genetic mutations. Specifically, for genetic diseases affecting tissues such as the liver, lungs, muscle, eyes, and ears, genome editing presents a direct method for repairing gene defects at the DNA level. In this section, we first elucidate the pathophysiology of genetic disorders affecting various organs. As summarized in Table 1, we provide an overview of the mutated genes, pathogenic causes, and affected organs associated with different genetic diseases. We then discuss current research on gene editing therapies for these genetic disorders. Specifically, as shown in Table 2, we summarize key clinical trials that have been conducted for these therapies.

### 3.1 Genetic diseases associated with liver dysfunction

The liver plays a critical role in various metabolic pathways, making it the source of many inherited metabolic liver diseases. A common characteristic among these disorders is a deficiency in the synthesis or functionality of proteins crucial to metabolic biochemical pathways^83^. Furthermore, the liver functions as a protein manufacturing facility, releasing a majority of the abundant proteins present in the bloodstream. This attribute positions the liver as a promising bioreactor for producing recombinant proteins and treating some genetic disorders, such as Familial hypercholesterolemia (FH) and hereditary angioedema (HAE)^76^. Conventional enzyme replacement therapy is available for only a few liver-related genetic diseases. Moreover, organ donor shortages restrict orthotopic liver transplantation, which necessitates lifelong immunosuppression. Consequently, strategies involving gene editing targeted at the liver have emerged as promising alternatives for treating genetic diseases associated with hepatic dysfunction.

#### 3.1.1 Tyrosinemia and genome editing strategies

HT1 is a rare autosomal recessive disease with a global incidence of approximately 1 in 100,000 to 120,000 newborns^84^. HT1 is characterized by a deficiency of fumarylacetoacetate hydrolase (FAH), the final enzyme in the tyrosine catabolic pathway. Disruption of this metabolic pathway leads to the accumulation of maleylacetoacetic acid and fumarylacetoacetic acid, resulting in significant hepatocyte injury due to their hepatotoxic nature^84^. P-hydroxyphenylpyruvate dioxygenase (HPD) is another enzyme involved in the tyrosine degradation pathway. Although mutations in the *HPD* gene are not directly responsible for HT1, they play a crucial role upstream of the FAH-catalyzed step. Inhibition of HPD reduces production of toxic metabolites downstream, thereby alleviating symptoms and disease progression^84^. Experimental treatments for HT1 have utilized *ex vivo* and *in vivo* gene editing techniques. In *ex vivo* treatments, hepatocytes are harvested from the donor, genetically modified with the CRISPR-Cas system to correct point mutations within the *Fah* gene, and then transplanted back into the animal model^85^. Furthermore, HT1 has been successfully cured in *Fah*^−/−^ mouse models through *in vivo* gene editing experiments utilizing BE or CRISPR-Cas9 systems^47^^,^^86^, ^87^, ^88^.

#### 3.1.2 FH

FH is a hereditary disorder characterized by abnormally high levels of low-density lipoprotein-cholesterol (LDL-C) in the blood. It occurs in two forms, heterozygous and homozygous^89^. In most cases, FH results from loss-of-function variations in the LDL receptor (LDLR) gene, including splicing mutations, large-scale DNA copy number variations, insertions and deletions, as well as missense and nonsense variants^89^. Mutations in ApoB also contribute to FH since ApoB serves as an LDLR ligand located on the surface of LDL particles. Approximately 2%–5% of individuals exhibiting the FH phenotype have loss-of-function variants in ApoB^90^. Additionally, Gain-of-function variants in the *PCSK9* gene, which inhibit LDLR, contribute to 1%–2% of FH cases^91^. Preclinical studies have investigated gene editing therapies for FH by targeting the above three genetic factors. Zhao et al.^92^ utilized AAV-CRISPR-Cas9 to correct the point mutation in *Ldlr*^*E208X*^ mice. Vanhoye et al.^93^ employed CRISPR-Cas9 to create *APOB* knockout (KO) and *APOB-p.Leu351Arg* knock-in (KI) Huh7 cells, reducing APOB expression. CRISPR-Cas9 and ABE systems have also been used to downregulate PCSK9 levels, showing promising results in FH treatment^48^^,^^94^, ^95^, ^96^.

#### 3.1.3 Transthyretin amyloidosis

Transthyretin amyloidosis (ATTR amyloidosis) is a severe disorder primarily characterized by neuropathy and/or cardiomyopathy^97^, ^98^, ^99^, occurring in both hereditary and wild-type forms^100^. The Transthyretin (TTR) is a 55 kDa tetrameric protein that plays a crucial role in the transportation of thyroxine and retinol-binding protein. Consequently, it contributes to nerve regeneration, behavior, cognition, and other essential functions^98^^,^^101^. The *TTR* gene is located on chromosome 18q12.1. More than 100 different mutations in the *TTR* gene have been identified as causative factors for hereditary ATTR amyloidosis^100^. Single-point mutations can induce misfolding and aggregation of TTR in the heart, nerves, and other tissues, leading to disease manifestation^98^. Considering the demonstrated benefits of TTR knockdown through siRNA-based and ASO-based clinical studies for patients with ATTR amyloidosis, CRISPR-Cas-mediated gene editing holds promise for its treatment. Furthermore, CRISPR-Cas technology enables precise targeting and editing of hepatocytes responsible for nearly all plasma TTR production while mitigating off-target effects. Currently underway is a Phase III trial (NCT06128629) investigating NTLA-2001 (Intellia Therapeutics), a CRISPR-based gene editing candidate that targets the *TTR* gene specifically for treating ATTR amyloidosis.

#### 3.1.4 HAE

HAE is a rare autosomal dominant disorder characterized by sporadic and recurrent episodes of non-urticarial swelling in the skin, gastrointestinal tract, upper airway, face, and throat^102^. These symptoms can significantly impair daily activities, work responsibilities, and recreational pursuits. Furthermore, laryngeal swelling poses a life-threatening risk^103^. The majority of HAE cases are associated with insufficient activity of C1 inhibitor (C1INH). The *SERPING1* gene encodes the C1INH protein, which is primarily synthesized in the liver and released into the bloodstream. C1INH serves as a key regulator for several proteases involved in the complement system, contact system, coagulation, and fibrinolysis pathways. Inadequate regulatory function of C1INH within the contact system leads to uncontrolled activation of factor XII (factor XIIa) and plasma kallikrein, resulting in excessive production of bradykinin—a vasoactive peptide^102^^,^^104^. NTLA-2002 (Intellia Therapeutics), an experimental CRISPR-Cas9-based therapy targeting liver cells, aims to disrupt the prekallikrein (*KLKB1*) gene responsible for its production. Currently undergoing a phase 1/2 clinical trial among adults diagnosed with type I or II HAE (NCT05120830), we will discuss this treatment later.

#### 3.1.5 PKU

PKU is a hepatic disorder characterized by a deficiency of the enzyme phenylalanine hydroxylase (PAH), leading to the accumulation of phenylalanine (L-Phe) in the bloodstream. This disease follows an autosomal recessive inheritance pattern, requiring mutations in both *PAH* alleles for its manifestation^105^. The elevated L-Phe level in the brain negatively affects neuropsychological function^105^. Current therapeutic approaches for PKU can be broadly categorized into two main strategies: (1) dietary restriction of phenylalanine intake and (2) pharmacological interventions like sapropterin or pegvaliase to control the disease^106^. However, both therapies face challenges in achieving optimal therapeutic outcomes, highlighting the need for a lifelong treatment solution for PKU. Gene editing therapy for PKU aims to correct the *PAH* gene or integrate a PAH expression cassette into the genome to restore normal enzyme function and metabolic balance. Although CRISPR-Cas9 has been used to co-deliver a DNA repair template with AAV vectors in mouse models, it failed to fully correct Phe levels^107^^,^^108^. However, studies using BEs or PEs showed better outcomes. For example, one study achieved a 41% *PAH* gene correction with an rAAV vector^109^. These new gene editing technologies may pave the way for the clinical treatment of PKU.

### 3.2 Genetic diseases associated with lung dysfunction

Lung genetic disorders primarily include various respiratory syndromes characterized by airway obstruction. Among these lung genetic disorders, CF, resulting from single-gene mutations, emerges as the most prevalent and severe disease^110^^,^^111^. In addition to CF, surfactant protein B (SP-B) deficiency also represents an autosomal recessive disorder^112^. These life-threatening diseases still lack effective treatment options. Recently, the use of CRISPR-Cas9 for precise gene editing has opened up new possibilities for treating lung genetic disorders^113^.

#### 3.2.1 SP-B deficiency and genome therapy strategies

SP-B deficiency is an autosomal recessive disorder, occurring in ∼1/1,000,000 births. Newborns with SP-B deficiency experience rapid respiratory deterioration, leading to mortality within 3 to 6 months without lung transplantation. The majority of affected infants inherit biallelic null variants of the *SFTPB* gene in a recessive manner, resulting in the complete or near-complete loss of mature SP-B expression^114^. In rare cases, extended survival beyond the first months or years has been associated with hypomorphic missense variants that allow partial expression and function of SP-B^115^. These findings underscore the urgent need to develop alternative therapeutic strategies for treating SP-B deficiency. Potential therapies, including *SFTPB* mRNA therapy^116^ and *SFTPB* cDNA transfection^117^^,^^118^, have demonstrated efficacy in extending the survival of SP-B deficient mice.

Additionally, gene editing techniques have been utilized to treat SP-B deficiency in mouse models. For example, Mahiny et al.^119^ effectively corrected SP-B deficiency by facilitating HDR and enabling sustained expression of SP-B using nuclease-encoding mRNA in a transgenic mouse model. However, gene therapies for treating SP-B deficiency remain in the preclinical stage and require further development.

#### 3.2.2 CF and gene editing strategies

CF is an inherited disorder caused by mutations in the *CFTR* gene, which encodes the cystic fibrosis transmembrane conductance regulator (CFTR) protein, essential for chloride and bicarbonate ion transport across epithelial cell membranes^110^^,^^111^. CF patients primarily suffer from recurrent lung infections and inflammations, which can lead to airway blockages and severe respiratory issues^110^. Due to its genetic complexity, over 2000 CFTR variants have been identified, with approximately 700 confirmed as disease-causing^120^. The F508del mutation, present on at least one allele in around 85% of CF patients, disrupts protein folding and membrane expression, significantly reducing the functional activity of CFTR channels. Other common mutations, including W1282X, R334W, and A455E, are also associated with multiple defects^121^. Gene editing advancements have shown promise in treating CF^113^. ABEs and PEs have repaired various nonsense mutations, including F508del, using epithelial cells or patient-derived intestinal organoids, restoring CFTR functionality^122^, ^123^, ^124^. A recent study by Sun et al.^125^ demonstrated the potential of LNPs to correct the R553X mutation in CF mouse lung stem cells, indicating the potential of *in vivo* targeted gene editing to cure CF.

### 3.3 Other genetic diseases

#### 3.3.1 Duchenne muscular dystrophy (DMD) and genome editing strategies

DMD is a debilitating X-linked recessive disorder caused by a genetic deficiency of dystrophin, a protein encoded by the *DMD* gene. Dystrophin primarily protects muscles from contractile damage and participates in the MARK2 kinase signaling process through its interactions with the dystrophin-associated protein complex (DAPC). This interaction regulates muscle satellite (stem) cell polarity and asymmetric division^126^^,^^127^. The absence of dystrophin disrupts the DAPC, leading to the mislocalization and reduced expression of its components from the sarcolemma^128^. Located within one of the largest known human genes spanning approximately 2.2 Mb and containing 79 exons, the DMD gene exhibits a high frequency of *de novo* mutations. Predominant mutations causing DMD include whole exon deletions (68%), exon duplications (11%), and nonsense mutations (10%)^129^.

The CRISPR-Cas system has facilitated promising therapeutic strategies for the genetic correction of DMD, focusing on exon excision, exon reframing, exon skipping, and precise point mutation correction^130^, ^131^, ^132^. The first CRISPR clinical trial for DMD, CRD-TMH-001 (NCT05514249), aimed to activate dystrophin expression *via* CRISPR activation (CRISPRa). CRISPRa utilizes catalytically inactive Cas9 (dCas9) fused with an effector domain, such as VP64, to enhance transcriptional activation without generating DSBs^133^. However, the trial was halted after the sole participant experienced fatal complications, possibly due to delivery efficiency, immune responses against AAV vectors, or off-target effects.

#### 3.3.2 Mucopolysaccharidoses and genome editing strategies

Mucopolysaccharidoses are inherited metabolic disorders caused by deficiencies in lysosomal enzymes essential for glycosaminoglycan (GAG) degradation. These disorders are classified into eleven types based on specific enzyme deficiencies outlined in an earlier review^134^. MPS I accounts for approximately 15% of mucopolysaccharidoses cases and results from a deficiency in alpha-(IDUA), which is responsible for the breakdown of GAGs such as heparan sulfate (HS) and dermatan sulfate (DS)^135^^,^^136^. GAG accumulation in lysosomes disrupts cellular function and tissue integrity, leading to diverse clinical manifestations^136^. The *IDUA* gene mutations in MPS I exhibit variable locations, with the three predominant variants identified as p.Trp402Ter, p.Gln70Ter, and p.Pro533Arg^134^. MPS II (Hunter Syndrome) is caused by a deficiency in the enzyme iduronate-2-sulfatase (IDS), which is also responsible for degrading GAGs in lysosomes. The main clinical symptoms of MPS II closely resemble those of MPS I^137^. MPS II is the only X-linked form of MPS (Xq28), with 658 variants identified in the *IDS* gene as of 2020. The most common variants include p.Arg468Gln, p.Arg468Trp, and p.Ser333Leu^134^.

Gene editing approaches utilizing ZFNs for the treatment of mucopolysaccharidoses are currently undergoing clinical trials^134^. The CHAMPIONS phase 1/2 clinical trial (NCT03041324) explored the ZFN-mediated introduction of a functional *IDS* transgene into the albumin locus *via* AAV2/6 in MPS II^138^. Despite the approach, post-treatment plasma IDS levels in participants remained relatively low. The “EMPOWERS” trial (NCT02702115) for MPS I employs a similar strategy, substituting *IDUA* for *IDS*, but plasma IDUA levels in participants showed no significant improvement^139^. These findings suggest that *in vivo* gene editing for mucopolysaccharidoses requires further optimization.

#### 3.3.3 Retinal and vitreoretinal diseases and genome editing strategies

Monogenic retinal and vitreoretinal diseases, such as LCA, night blindness, retinitis pigmentosa, and achromatopsia^140^, are ideal for CRISPR applications in the eye. The small size of the eye reduces the need for extensive vector production, while its enclosed structure limits the spread of vectors, thereby enhancing safety. The immune-privileged nature of the eye may support prolonged expression of prokaryotic Cas proteins^141^. Gene editing strategies for inherited eye disorders typically involve gene replacement or augmentation to address recessive or loss-of-function mutations, while gene inactivation or repression is employed for dominant or gain-of-function mutations^142^. Preclinical therapeutic strategies utilizing CRISPR-Cas systems have been applied to treat various eye diseases. A detailed discussion of these studies is beyond the scope of this review, but can be found in other comprehensive reviews^143^.

LCA, a genetic retinal disorder that typically presents in early childhood, can lead to blindness. EDIT-101 (Editas Medicine), a gene editing candidate for LCA, utilized an AAV5 vector containing expression cassettes for Cas9 and a pair of sgRNAs designed to correct aberrant splice donor sites and restore normal *CEP290* expression^16^. Phase 1/2 clinical trial (NCT03872479) results indicated that EDIT-101 was well-tolerated, with no significant adverse events, and 8/14 participants reported symptom improvement^144^.

#### 3.3.4 Hearing loss (HL) and genome editing strategies

HL is the predominant sensory disorder affecting humans, with a global prevalence of over 5%, equivalent to 466 million individuals. It is projected that by 2050, this number will exceed 900 million, affecting ∼10% of the population^145^. HL can be congenital or acquired and may result from genetic or environmental factors, or a combination of both. For example, congenital HL affects approximately 1 in 500 newborns, with more than half of these cases attributed to genetic factors (genetic HL), while the remaining cases are caused by environmental factors (non-genetic/acquired HL)^146^. In recent years, the CRISPR-Cas system has gained significant attention as a promising approach for both modeling and treating genetic HL. Different forms of CRISPR-Cas cargos (DNA, mRNA, and protein) have been introduced into inner ear cells using viral^147^^,^^148^ and non-viral^149^^,^^150^ delivery methods. Furthermore, considering that more than 80% of deafness-associated variants are single-nucleotide variants (SNVs), BEs hold great potential as tools for the treatment of genetic HL^145^. For example, Xue et al.^151^ utilized an enhanced mini-dCas13X RNA base editor (emxABE) to edit the *OTOF* gene at the RNA level by converting the TAG stop codon to TGG. This intervention successfully restored otoferlin expression and auditory function in a mouse model of genetic HL.

#### 3.3.5 Fragile X syndrome (FXS) dystrophy and genome editing strategies

FXS is classified as an intellectual disability and autism spectrum disorder, with a prevalence of approximately 2.1% among individuals with autism spectrum disorders (ASDs)^152^^,^^153^. The condition is characterized by an excessive expansion of CGG triplet repeats in the 5′ untranslated region of the X-linked fragile X mental retardation 1 gene (*FMR1*), leading to the loss of fragile X mental retardation protein (FMRP)^154^. FMRP is an RNA-binding protein that plays a crucial role in regulating the synthesis of numerous proteins essential for synaptic function, particularly group 1 metabotropic glutamate receptors (mGluRs) and GABA receptors^155^. Consequently, the loss of FMRP will subsequently impair brain development. Preclinical studies have utilized CRISPR-based gene editing systems to delete or modify CGG repeats of the *FMR1* gene^153^^,^^156^^,^^157^. Park et al.^156^ utilized the CRISPR-Cas9 gene editing tool to induce DSBs upstream of the CGG repeat region, thereby reactivating silenced gene expression and promoting FMRP production.

#### 3.3.6 Sickle cell disease (SCD) and genome editing strategies

SCD is the most prevalent monogenic hematologic disorder, characterized by congenital hemolytic anemia resulting from an inherited point mutation in the *β*-globin gene on chromosome 11^158^. This mutation results in the substitution of valine for glutamate at the sixth codon of the hemoglobin beta-chain (*HBB*), producing abnormal hemoglobin S (HbS). Under hypoxic or acidic conditions, HbS polymerizes, causing red blood cells (RBCs) to deform into a narrow sickle shape^159^^,^^160^. This change in RBC morphology reduces their flexibility and lifespan, leading to recurrent vascular occlusion, acute and chronic pain, chronic hemolytic anemia, increased susceptibility to infections, and multi-organ failure^161^. Treatment options for this condition have remained limited. Medications for SCD only manage and alleviate symptoms but do not provide a cure. CRISPR-Cas9 gene editing offers a promising alternative. Initially applied *in vitro* to correct mutated genes in primary bone marrow-derived CD34^+^ cells from SCD patients, this technology has already been implemented in a clinical trial (NCT05456880)^162^. Recent studies have utilized animal models to assess the correction rates of various methods *in vivo*^162^.

## 4 Non-viral gene editing delivery for treating genetic diseases

**Figure 2:**
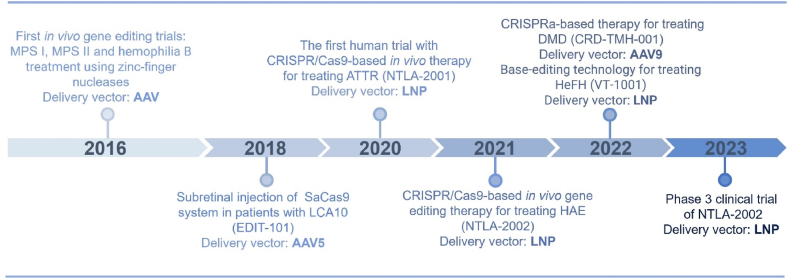
A timeline of essential processes in treating genetic diseases with *in vivo* gene editing technologies. AAV vectors were used in early gene therapy trials, starting in 2016, for diseases such as MPS I, MPS II, and hemophilia B. They were later used in the 2018 subretinal injection for LCA10 patients (EDIT-101), and also for treating DMD in 2022. However, LNPs started appearing prominently in 2020 with the first human trial using CRISPR/Cas9-based *in vivo* therapy for treating ATTR. LNPs were then also used for treating HAE (NTLA-2002) in 2021 and are currently involved in Phase III trials for NTLA-2002 in 2023.

Non-viral gene therapy approaches offer promising solutions to the challenges posed by viral vectors. These synthetic vectors are typically less immunogenic, can accommodate larger genetic payloads, and are easier to manufacture. Consequently, there is significant interest in developing effective and safe non-viral methods for delivering genome editing enzymes to treat genetic diseases. In the field of genetic disease therapy, numerous non-viral systems, including inorganic vectors, polymeric vectors, lipid-based vectors, and biomimetic vectors, have undergone extensive investigation in both preclinical and clinical settings (Table 3^47^, ^48^, ^49^^,^^82^^,^^88^^,^^94^^,^^100^^,^^119^^,^^125^^,^^135^^,^^153^^,^^163^, ^164^, ^165^, ^166^, ^167^, ^168^, ^169^, ^170^, ^171^, ^172^, ^173^, ^174^, ^175^, ^176^, ^177^, ^178^, ^179^). Notably, with improvements in LNP delivery systems, several ongoing non-viral clinical trials targeting genetic diseases are being conducted (Fig. 2). The following sections will provide a comprehensive overview of how these four types of vectors facilitate the delivery of gene editing tools for treating genetic disorders.

**Table 3:** Non-viral delivery strategies for delivering gene editing machinery to treat genetic diseases.

Delivery vector	Genetic disease	Gene editing format	Administration route	Ref.
Chitosan and AAV	SP-B deficiency	*ZFN* mRNA and DNA donor	Intratracheal injection	119
Polymeric nanocapsules	Eye diseases	spCas9 RNP	Subretinal injection	163
Gold nanoparticles	DMD	Cas9 RNP and donor DNA	Intramuscular injection	164
	FXS	Cas9 RNP; Cas12a RNP	Stereotaxic injection to brain	153
Cationic liposomes	HL	TALENs; Cas9 RNP	Inner ear injection	165
		spCas9 RNP	Inner ear injection	166
	MPS I	CRISPR-Cas9 pDNA and DNA donor oligo/plasmid	Intravenous injection	135
Lipid nanoparticles and AAV	HT1	spCas9 mRNA, sgRNA and donor DNA	Intravenous injection	88
Lipid nanoparticles	HT1	*ABE* mRNA and sgRNA	Intravenous injection	47
	FH	spCas9 mRNA and two sgRNAs;	Intravenous injection	94
		*ABE* mRNA and sgRNA	Intravenous injection	48,167
		*ABE* mRNA and gRNA	Retro-orbital injection	168
	ATTR amyloidosis	*Cas9* mRNA and sgRNA	Intravenous injection	100,169,170
	DMD	Cas9 RNP	Intravenous injection	82
	PKU	*CBE* mRNA and sgRNA	Intravenous injection	171
		ABE RNP	Retro-orbital injection	172
	HAE	*Cas9* mRNA and sgRNA	Intravenous infusion	173
	CF	*ABE8e* mRNA and sgRNA	Intravenous infusion	125
	SCD	*Cas9* mRNA and sgRNA, ABE8e mRNA and sgRNA	Intravenous infusion	174
	Eye diseases	ABE RNP and PE RNP	Subretinal injection	175
Virus-like particles	DMD	spCas9 RNP	Intramuscular injection	176
		Cas9 RNP; ABE RNP	Intramuscular injection	49
	AMD	*Cas9* mRNA and sgRNA	Subretinal injection	177
	LCA 10	ABE RNP	Subretinal injection	178
	HT1	spCas9 RNP	Injection in embryos; retro-orbital injection	179

Abbreviations: AAV, adeno-associated virus; ABE, adenine base editor; AMD, age-related macular degeneration; ATTR, transthyretin amyloidosis; CBE, cytosine base editor; Cas9, CRISPR-associated protein 9; Cas12a, CRISPR-associated protein 12a; CF, cystic fibrosis; DMD, Duchenne muscular dystrophy; FH, familial hypercholesterolaemia; FXS, fragile X syndrome; gRNA, guide RNA; HAE, hereditary angio-oedema; HL, hearing loss; HT1, hereditary tyrosinaemia type 1; LCA 10, Leber congenital amaurosis type 10; LNP, lipid nanoparticle; MPS I, mucopolysaccharidosis type I; pDNA, plasmid DNA; PE, prime editor; PKU, phenylketonuria; RNP, ribonucleoprotein; SCD, sickle cell disease; sgRNA, single-guide RNA; spCas9, *Streptococcus pyogenes* Cas9; TALENs, transcription activator-like effector nucleases; ZFN, zinc-finger nuclease.

### 4.1 Inorganic vectors

Inorganic nanoparticles, such as GNPs, have been explored as a versatile platform for nucleic acid delivery and imaging in various applications. They can be precisely engineered to efficiently target and deliver therapeutic cargos^180^. Moreover, emerging research suggests that the immune responses triggered by nanoparticles can be either enhanced or inhibited, with their immune compatibility being significantly influenced by their surface chemistry^181^. Therefore, optimizing the composition, size, architecture, zeta potential, and surface coating of inorganic nanoparticles holds great promise for improving their compatibility.

**Figure 3:**
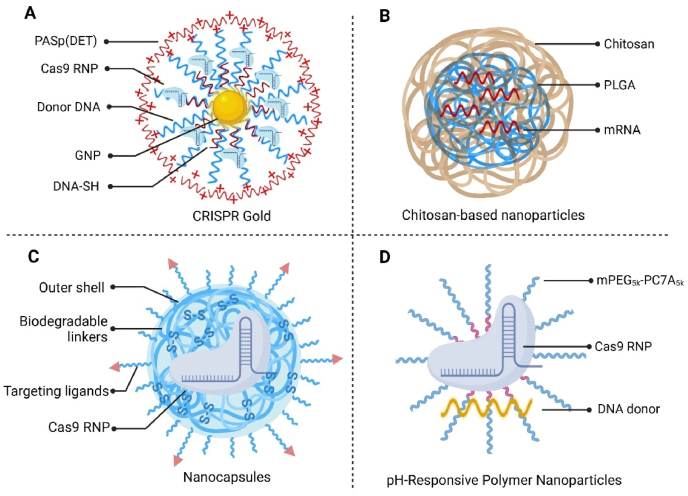
Inorganic and polymeric gene editing delivery systems. (A) The CRISPR-Gold system for delivering Cas9 RNP and donor DNA. DNA-SH was conjugated with 15-nm-diameter GNPs, which were then hybridized with single-stranded donor DNA. After loading the Cas9 and gRNA, the silicate and PAsp(DET) coatings were applied. (B) The chitosan-based delivery system utilizes chitosan-coated PLGA nanoparticles loaded with modified mRNA to deliver ZFN nucleases efficiently. (C) Biodegradable polymeric nanocapsules contain biodegradable disulfide crosslinkers sensitive to GSH, crosslinkers interacting with Cas9 RNP, and outer biodegradable liners. (D) The pH-sensitive mPEG-PC7A could form nanoparticles through self-assembly, packing donor DNA and Cas9 RNP to build HDR nanoparticles.

GNPs are monodisperse nanostructures that can effectively deliver macromolecules such as proteins, pDNA, and siRNA through electrostatic interactions by functionalizing them with various ligands and chemical moieties (Fig. 3A)^182^^,^^183^. As versatile delivery platforms, GNPs offer several advantages: (i) GNPs can be easily fabricated in a scalable manner with minimal size variation, (ii) they can simultaneously carry multiple components by creating multilayers, with each monolayer capable of accommodating large quantities of cargo, and (iii) their biodistribution can be tailored by modifying their size and surface properties^182^^,^^183^.

#### 4.1.1 CRISPR-gold for the treatment of DMD and FXS dystrophy

Lee et al.^164^ developed “CRISPR-Gold”, using GNPs to co-deliver Cas9 RNP and donor DNA for the mutation correction in DMD mice. As shown in Fig. 3A, the CRISPR-Gold system encapsulates gene editing components by interaction between GNPs and thiol-terminated DNA to enable subsequent hybridization with donor DNA. Subsequently, Cas9 RNPs were non-covalently attached to the particles, and a negatively charged silica layer was deposited onto the nanoparticles to form complexes with the cationic polymer poly(aspartamide) bearing 1,2-diaminoethane (PAsp (DET)). PAsp (DET) exhibited distinct conformations under neutral and acidic conditions, allowing for endosomal disruption, and its weak proton sponge effect facilitated endosomal escape^184^. In primary myoblasts from DMD mice, CRISPR-Gold achieved a 3.3% HDR efficiency in correcting point mutations in the dystrophin gene and restored 0.3% of wild-type dystrophin expression. Intramuscular injection in DMD mice led to a 5.4% correction of the dystrophin gene, and robust dystrophin protein was observed in muscle tissues following *in vivo* treatment. This strategy highlights the potential of HDR in restoring dystrophin without the disruptions caused by NHEJ processes.

In 2017, Staahl et al.^185^ demonstrated non-viral delivery of Cas9 RNPs for *in vivo* gene editing in multiple brain regions by fusing NLS to the N- and C-termini of the Cas9 protein. Expanding on this, Lee et al.^153^ employed CRISPR-Gold for precise gene editing in the brain, specifically targeting the *mGluR5* gene (*Grm5*) in the striatum of a mouse model with FXS. By stereotaxic injection of CRISPR-Gold loaded with Cas9 or Cas12a RNPs, they achieved a 40%–50% reduction in mGluR5 expression in *Fmr1* KO mice, rescuing excessive digging and jumping behaviors. This study underscores the potential of CRISPR-Gold in treating brain disorders and paves the way for future advancements.

Compared to other inorganic vectors such as silica nanoparticles (SiNPs), GNPs exhibit unique advantages in delivering genome editors, including enhanced cellular internalization and versatile functionalization. However, their medical applications face significant challenges. First, unlike biodegradable SiNPs, which are metabolized into biocompatible byproducts, GNPs are non-biodegradable and may accumulate *in vivo*, posing long-term toxicity risks^186^. Second, while GNPs are generally non-toxic at low concentrations, significant cytotoxicity is observed at concentrations above 25 μmol/L^187^. Third, GNPs, especially of smaller sizes, tend to accumulate in healthy organs enriched with the reticuloendothelial system (RES), such as the liver, spleen, or in RES-depleted organs like the kidneys, leading to cytotoxic effects^188^^,^^189^. Finally, acute toxicity triggered by GNPs, including inflammation and apoptosis, has been reported in preclinical studies^190^^,^^191^. Consequently, limited commercial opportunities have been generated for their utilization in the medical field.

### 4.2 Polymeric vectors

#### 4.2.1 Chitosan for treatment of SP-B deficiency

Chitosan is a biodegradable polymer composed of repeating d-glucosamine and *N*-acetyl-d-glucosamine units, exhibiting favorable biocompatibility, non-toxicity, and biodegradability^192^. The primary amine groups present in each d-glucosamine unit can be protonated under slightly acidic pH conditions, enabling chitosan to interact with negatively charged nucleic acids and form nano-sized complexes^193^. These interactions facilitate the efficient encapsulation and delivery of nucleic acids within chitosan-based systems.

In a pivotal investigation, chitosan-coated poly (lactic-*co*-glycolic acid) (PLGA) nanoparticles complexed with *ZFN* mRNA and AAV6 carrying a DNA template donor were (Fig. 3B) co-administered to the lungs of SP-B deficient mouse model^119^. This was the first use of non-viral materials for *in vivo* delivery of an endonuclease gene editing system, albeit with assistance from AAV. SP-B deficiency is a fatal disorder primarily affecting infants, where lung transplantation remains the only available treatment option^194^. As this autosomal recessive condition results from mutations in the *SFTPB* gene, gene editing offers a potential cure^195^. In this investigation, *ZFN* mRNA was loaded onto chitosan-coated PLGA nanoparticles at a weight ratio of 25:1, achieving significantly higher ZFN protein expression compared to naked mRNA delivery. Co-delivery of AAV6 with *ZFN* mRNA-nanoparticles resulted in approximately 28% indels in lung cells and approximately 11% HDR efficiency as compared to approximately 21% indels and 8% HDR achieved when AAV6 was co-delivered solely with *ZFN* mRNA alone, highlighting the ability of nanoparticles to facilitate efficient gene editing^119^.

Recent investigations have focused on enhancing the delivery of CRISPR-Cas9 systems using chitosan. One approach involves conjugating polyethylene glycol (PEG) to chitosan, improving performance^192^. Another strategy incorporates negatively charged moieties like additional NLSs to address release-related challenges^196^. These studies indicate that chitosan nanoparticles hold potential for enhanced safety^55^ and efficiency^119^^,^^192^^,^^196^.

However, chitosan faces inherent limitations in gene delivery efficacy due to its high molecular weight, which reduces solubility, compromises cell-specific targeting, diminishes endosomal buffering capacity, and hinders the intracellular dissociation of nucleic acid payloads. To overcome these challenges, structural or chemical modifications are essential for optimizing functionality. Moreover, clinical trials utilizing such vectors have not yet been reported, highlighting the need for further modifications to improve delivery efficiency.

#### 4.2.2 Polymeric nanocapsules (NCs) for treatment of eye diseases

Nanoparticle technologies provide several advantages to protein delivery, including protection from premature degradation or denaturation in biological environments and improving the systemic circulation half-life of proteins with poor pharmacokinetic properties. They also enable controlled, sustained, or tunable release, maintaining drug concentrations within the therapeutic range, and facilitating targeted delivery to diseased tissues, cells, and intracellular compartments. Consequently, these advancements enhance the safety and efficacy of biologic therapeutics^197^^,^^198^.

To achieve stable and efficient delivery of Cas9 RNPs both *in vitro* and *in vivo*, Chen et al.^163^ developed NCs through *in situ* free-radical polymerization (Fig. 3C). NCs consisted of six key components, including cationic and anionic monomers for charge adjustment, imidazole monomers to facilitate endosomal escape, biodegradable disulfide crosslinkers sensitive to intracellular glutathione (GSH), acrylate PEG, and acrylate-PEG-ligand for interaction with RNPs, as well as outer-shell targeting ligands. The polymerization began by mixing sNLS-Cas9-sNLS protein with sgRNA, followed by dilution in sodium bicarbonate buffer and sequential monomer additions. Surface modification with all-*trans* retinoic acid (ATRA) enhanced cellular uptake of nanoparticles *in vivo*. Gene editing potential was tested using Ai14 mouse models, where ATRA-NCs achieved over 4% fluorescence expression in RPE cells, compared to 1% by non-ATRA NCs after 12 days of subretinal injection. NCs also restored approximately 2% *tdTomato* expression in the basal lamina following intramuscular injection, whereas unencapsulated RNPs failed to induce gene deletion. In conclusion, polymeric NCs efficiently load and release RNPs, and their ability to incorporate various outer-shell ligands makes them highly adaptable for treating genetic eye and muscle diseases.

#### 4.2.3 pH-responsive nanoparticles for the treatment of DMD

HDR-based gene correction holds promise for treating a substantial portion of DMD patients, as approximately 30% of them exhibit single-base mutations or small deletions in the dystrophin gene. However, the co-delivery of Cas9 RNP and DNA templates presents a challenge due to their distinct morphologies and surface charges. Therefore, it is essential to develop a vector capable of interacting with both Cas9 RNP and DNA donor molecules.

Xie et al.^199^ addressed this issue by selecting an amphiphilic copolymer, methoxy-poly(ethylene glycol)-*b*-poly(2-(azepan-1-yl)ethyl methacrylate) (mPEG-PC7A), which exhibits protonated and deprotonated segments at physiological pH. This copolymer facilitated the delivery of single-strand oligonucleotides (ssOND) and Cas9 RNP into the cell nucleus (Fig. 3D). In HEK293 cells, both HDR-nanoparticles and NHEJ-nanoparticles achieved high efficacy in genome editing. In mdx mouse models harboring a mutation in exon 23 of the *DMD* gene with impaired dystrophin formation, these nanoparticles induced HDR in 2.5% and NHEJ in 8.6% of tibialis anterior muscles, leading to notable restoration of muscle strength as well. As highlighted by the authors, this delivery strategy exhibits potential for clinical translation due to its facile fabrication process and storage stability.

### 4.3 Lipid-based vectors

**Figure 4:**
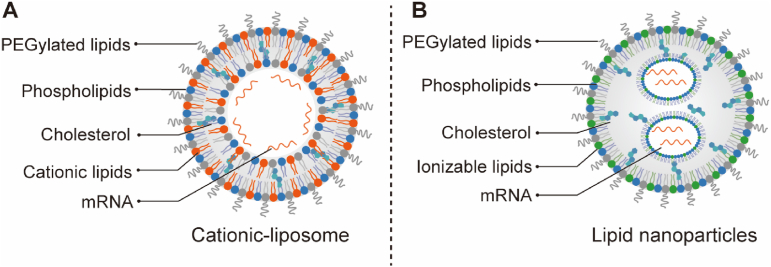
Structure of lipid-based vectors. (A) Cationic-liposomes. Cationic liposomes usually contain four components: PEGylated lipids, phospholipids, cholesterol, and cationic lipids. (B) Traditional LNPs have been applied in treating liver- and muscle-related genetic diseases. These LNP typically consist of PEGylated lipids, phospholipids, cholesterol, ionizable lipids, and mRNA.

The emergence of lipid-based vectors has opened new possibilities for the delivery of biological macromolecules in the biomedical field^200^, ^201^, ^202^, ^203^, ^204^. Liposomes, which are closed lipid bilayer vesicles, have been among the earliest nanomedicine delivery systems utilized in clinical settings (Fig. 4A)^205^. More recently, LNPs have gained attention as an upgraded version of liposomes. LNPs typically consist of four key components (Fig. 4B)^206^: (i) Ionizable lipids enable efficient encapsulation of cargos, enhance cellular uptake *via* charge-mediated membrane interactions, and facilitate endosomal escape for intracellular release. (ii) Zwitterionic phospholipids stabilize LNPs by preventing aggregation and balancing surface charge to reduce serum protein interactions, boosting delivery efficiency. (iii) PEGylated lipids extend blood circulation *via* a protective hydrophilic layer that minimizes immune clearance and controls nanoparticle size for optimized biodistribution. (iv) Cholesterol reinforces structural stability by filling phospholipid gaps and aiding membrane fusion^206^. Together, these components ensure LNPs act as biocompatible, efficient delivery vehicles with minimized therapeutic side effects.

With the advancement of gene editing technologies, cationic lipids and LNPs have successfully been employed for delivering gene editing tools in all three formats: pDNA, mRNA, and RNPs^201^.

#### 4.3.1 Cationic liposomes for the treatment of HL and MPS I

In 2014, Zuris et al.^165^ successfully edited the mouse inner ear using nuclease-encapsulated cationic liposomes. They enhanced electrostatic interactions by fusing super negatively charged green fluorescent protein (GFP) to Cre recombinase, TALENs, and Cas9, enabling effective delivery of gene editing proteins. Using commercial reagents RNAiMAX and Lipofectamine 2000 to deliver Cas9 RNPs, they achieved 65% and 80% gene KO in U2OS human cells. Injection of Cas9 RNP-lipid complexes into mouse inner ear models resulted in a 13% gene KO, potentially aiding hearing recovery in genetic ear disease models.

In a subsequent 2018 study, the same research team advanced cationic lipid-mediated Cas9 RNP delivery to address genetic hearing loss (HL)^166^. Genetic HL, which severely impacts language development and social interaction, often results from mutations in the transmembrane channel-like 1 (*TMC1*) gene, which plays a critical role in the formation of mechanically activated channels in hair cells necessary for sensory induction^207^. Gao et al.^166^ targeted the *TCM1* mutation in a *Tmc1*^*Bth/+*^ mouse model, which mimics human progressive HL due to a dominant mutation (p.M412K, c.T1235A). *In vitro* experiments involved coating spCas9 RNP with Lipofectamine 2000 and delivering the resulting complex into primary fibroblasts derived from mutant mice, leading to targeted indels reaching up to 10%. Comparing pDNA and RNP delivery, they found that RNPs had only one off-target site, while DNA transfection had nine, supporting the safety profile of RNPs. *In vivo* experiments entailed injecting complexes of Cas9 with sgRNA and Lipofectamine 2000 into the scala media of newborn mouse models. This resulted in 0.92% editing efficiency of *TMC1* genes in the organ of Corti, which contains hair cells, and significantly improved responsiveness to stimulation at 45 kHz after four weeks of treatment for mice. The study highlights the efficient delivery methods using lipids for gene editing tools within inner ear tissues, as well as establishes feasibility for correcting genetic deafness.

Schuh et al.^135^ addressed MPS I using cationic liposomal carriers to deliver CRISPR-Cas9, successfully correcting *Idua* mutations both *in vitro* and *in vivo*. They created lipids–CRISPR-Cas9 complexes using the film dispersion method, incorporating CRISPR-Cas9 DNA and an *Idua* donor oligo. In fibroblasts derived from MPS I patients, these complexes restored approximately 3% normal IDUA activity after a single transfection, with sustained effects observed for over 30 days. In more rigorous *in vivo* experiments, they injected the complexes into the superficial temporal vein of MPS I *Idua*^*KO*^ mice. The treated mice exhibited 4%–7% IDUA activity in the serum compared to normal mice, accompanied by reduced levels of GAGs. The study also showed improvements in cardiovascular parameters, such as left ventricular shortening fraction and systolic/diastolic diameter, which are critical since heart damage is a leading cause of mortality in MPS I. However, biodistribution studies revealed the presence of liposomal complexes in the heart, liver, lung, and spleen, indicating potential off-target effects.

In summary, cationic lipids have shown promise for *in vivo* gene editing. However, it is crucial to address certain limitations associated with these cationic particles, such as their positive charge and large size, which may lead to immune activation, hemolysis, and other toxic effects^200^. Exploring alternative approaches with better *in vivo* suitability is essential for advancing gene editing applications.

#### 4.3.2 LNPs for the treatment of liver diseases

Over the past two decades, there has been a consistent increase in mortality rates attributed to liver diseases, with a rise from 3% to 3.5% of total global deaths^208^. Consequently, there is a pressing demand for drug delivery systems that can selectively target diseased hepatic cells while enhancing the stability of therapeutic cargos. LNP technology exhibits promise for liver targeting and treatment of hepatic disorders. Notably, ONPATTRO, the first approved nanomedicine for liver targeting, is based on LNP architecture^209^. This section reviews LNPs used to treat genetic liver disorders such as HT1, FH, ATTR, and HAE.

To treat HT1, Yin et al.^88^ combined non-viral and viral vectors to deliver a CRISPR system along with a DNA template donor for localized gene correction. They used LNPs to deliver *Cas9* mRNA, enabling transient Cas9 expression, while utilizing AAV-delivered sgRNA and the HDR template to correct the *Fah* mutation. In 293T reporter cells, this approach achieved 80% frameshift *via* NHEJ and only a 13% off-target rate compared to lentiviral Cas9. *In vivo*, separate intravenous injections of HDR AAV and nano Cas9 in *Fah*^*mut/mut*^ mice resulted in approximately 6% of hepatocytes regaining Fah expression, with treated liver cells exhibiting 9.5% of wild-type *Fah* mRNA expression.

In another study, Song et al.^47^ used LNPs to deliver optimized mRNA encoding an ABE and sgRNA to restore FAH expression in diseased hepatocytes. As previously mentioned, ABEs are ideal for correcting G > A point mutations in *Fah*^*mut/mut*^ mice. The study showed that intravenous administration of LNP-ABE mRNA and LNP-sgRNA three to four times resulted in approximately 0.8% Fah^+^ hepatic cells 5 days after treatment. Although LNPs did not exhibit comparable efficiency to the alternative approach in this study, they nonetheless demonstrated the potential for non-viral delivery of ABEs in liver-based treatments and genetic disease correction.

PCSK9, crucial for modulating cholesterol levels, has gained attention in cardiovascular health. Gain-of-function mutations in *PCSK9* reduce LDLR expression, raising LDL-C levels^210^. Therefore, in 2017, Yin et al.^94^ used LNP complexes to edit the *PCSK9* gene in hepatocytes of C57BL/6 mice. The LNP formulation consisted of two chemically-modified sgRNAs and *Cas9* mRNA. Remarkably, they achieved approximately 83% editing efficiency of the *PCSK9* gene in the liver and undetectable serum PCSK9 levels after a single intravenous injection^94^. This study was the first to demonstrate a fully non-viral carrier for *in vivo* gene editing. Liu et al.^95^ later developed a biodegradable LNP formulation with disulfide bonds for efficient *Cas9* mRNA and sgRNA release, targeting hepatocytes to reduce PCSK9 levels by about 20%.

To address off-target risks and translate ABEs from laboratory to clinical applications, Rothgangl et al.^48^ injected ABE-encoding mRNA and PCSK9-targeting sgRNA in mice and macaques, achieving 50.9% editing efficiency in mice after a single injection and 67.3% after re-treatment. In macaques, the gene-editing system achieved approximately 27.6% on-target editing (A > G) in the *PCSK9* gene after a single dose, leading to a 26% reduction in serum PCSK9 levels and a 9% reduction in LDL levels compared to baseline. While no ABE-specific antibodies were detected in mice, they were found in macaques, possibly limiting further editing. These findings support the clinical potential of LNP-encapsulated ABEs for treating familial hypercholesterolemia. In another study, Musunuru et al.^96^ used LNPs with *ABE* mRNA and *PCSK9* gRNA to edit the *PCSK9* gene in cynomolgus monkeys, achieving a remarkable 90% reduction in PCSK9 and a 60% reduction in LDL-C levels in the blood. This led to the development of VERVE-101 (Verve Therapeutics), an LNP complex currently in clinical trial (NCT05398029). One year after administration, LDL-C was reduced by 68%, and blood PCSK9 level was reduced by about 89%^167^. These studies offer valuable insights into PCSK9 regulation for FH treatment and other cardiovascular diseases.

Standard LNPs utilize the LDLR-mediated endocytosis pathway for cellular entry, but this is limited in patients with homozygous familial hypercholesterolemia (HoFH) due to the lack of functional LDLR. Kasiewicz et al.^168^ investigated the delivery of the ABE system to the liver. GalNAc-LNPs effectively delivered the ABE system to the liver independent of LDLR, editing the *ANGPTL3* gene in *LDLR*-KO NHP models. A single administration of GalNAc-LNPs led to a remarkable 89% reduction in ANGPTL3 protein levels and a significant 35% decrease in blood LDL-C concentrations in the NHP model, suggesting GalNAc-LNPs as a promising tool for HoFH patients.

PKU is an autosomal recessive metabolic liver disease. Villiger et al.^50^ utilized AAV-based CBE systems to correct PKU mouse models, but due to AAV’s limited capacity, they employed a split CBE approach, successfully reducing blood L-Phe levels. Nevertheless, CBEs can induce severe off-target mutations, prompting a shift to transient expression using LNP-delivered *CBE* mRNA. Thus, another study proposed minimizing the duration of CBE as a solution to mitigate this problem^171^. The authors shifted their focus from AAV to LNPs and utilized mRNA formulation of CBEs for the transient expression. By delivering *CBE* mRNA and sgRNA using LNPs, transient gene editing achieved a 21% conversion from C > T in hepatocytes, with 10.8% of reads supporting PAH restoration after treating the PKU mice twice a week^172^.

**Figure 5:**
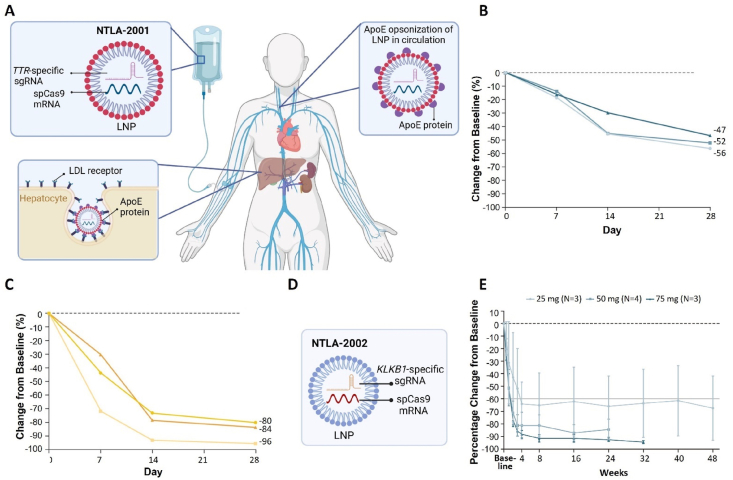
*In vivo* pharmacologic properties of NTLA-2001 and NTLA-2002 in patients. (A–C) Mechanism of action and *in vivo* pharmacologic properties of NTLA-2001^100^. (A) A schematic illustration depicts the primary components of NTLA-2001 and the transport of the NTLA-2001 LNP into hepatocytes. The active components of NTLA-2001 are an mRNA molecule encoding SpCas9 protein and an sgRNA molecule specific to the human gene encoding TTR. After intravenous administration of NTLA-2001 and its entry into the circulation, the LNP is opsonized by apolipoprotein E (ApoE) and transported directly through the systemic circulation into the liver. NTLA-2001 is then expected to undergo uptake by the LDLR, which is expressed on the surface of the hepatocytes. (B) Change in serum TTR concentration in patients who received 0.1 mg/kg NTLA-2001. (C) Change in serum TTR concentration in patients who received 0.3 mg/kg NTLA-2001. (D, E) Components and *in vivo* pharmacologic properties of NTLA-2002^173^. (D) A schematic illustration shows the primary components of NTLA-2002. (E) Change from baseline in the total plasma kallikrein protein level according to NTLA-2002 dose.

Silencing the *TTR* gene in hepatocytes is a promising therapeutic strategy for hereditary ATTR amyloidosis^169^. Finn et al.^170^ introduced an LNP-based approach for *TTR* editing using single particles complexed with *Cas9* mRNA and gRNA for simultaneous delivery. A single administration reduced serum TTR levels by over 97%, sustained for 12 months. NTLA-2001, further evaluated in NHPs, achieved over 60% liver editing and eliminated more than 95% of circulating TTR^169^. Encouraged by these results, NTLA-2001 entered clinical trial (NCT04601051), showing 52% and 87% reductions in serum TTR levels after low-dose and high-dose administrations, respectively (Fig. 5A–C)^100^. The Phase III study of NTLA-2001 has been initiated (NCT06128629), with further clinical results anticipated to bring this treatment closer to the bedside.

NTLA-2002 is a CRISPR-Cas9-based gene-editing therapy for HAE, targeting the KLKB1 gene in the liver to reduce plasma kallikrein levels and prevent angioedema attacks (Fig. 5D)^173^. Using liver-targeting LNP technology, NTLA-2002 encapsulates *Cas9* mRNA and sgRNA specific to the *KLKB1* gene, enabling precise genetic modifications in the liver. A Phase 1/2 clinical study (NCT05120830) demonstrated a favorable safety profile with significant reductions in plasma kallikrein levels and angioedema attack frequency. NTLA-2002 maintained plasma kallikrein reductions of 67% in the 25-mg group, 84% in the 50-mg group, and 95% in the 75-mg group. Angioedema attack frequency decreased by 91%, 97%, and 80% in the respective groups. LNP LP000001 was detectable in plasma for up to 15 days, underscoring the importance of LNPs in sustained NTLA-2002 delivery (Fig. 5E).

#### 4.3.3 SORT for the treatment of lung diseases

**Figure 6:**
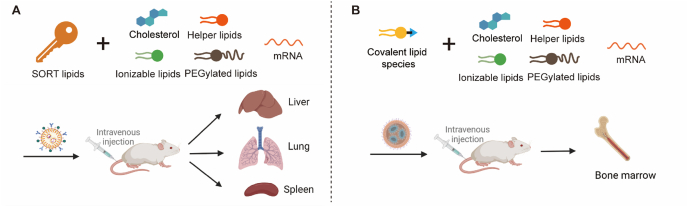
LNP engineering strategies for tissue-specific delivery. (A) Selective Organ Targeting (SORT) LNPs. Incorporating a fifth SORT molecule into a conventional four-component LNP redirects gene-editing cargos to the liver, lung, or spleen^211^. (B) Covalently cross-linked LNPs. Introducing a cross-linkable lipid into the five-component base formulation yields covalent LNPs that achieve broad bone-marrow mRNA delivery and genome editing across 14 hematopoietic cell types, including HSCs, progenitors, and immune cells^174^.

The selective organ targeting (SORT) strategy enables precise delivery of therapeutic cargos, including mRNA, *Cas9* mRNA/single guide RNA, and Cas9 RNP, to specific organs such as the lungs, spleen, and liver (Fig. 6A)^211^. To explore *in vivo* gene editing in lung stem cells for genetic lung diseases, such as CF, Sun et al.^125^ utilized lung-targeting SORT LNPs to deliver *ABE8e* mRNA and sgRNA for correcting the R553X mutation in CF mouse models. The researchers first co-encapsulated *ABE* mRNA and sgR553X to target the R553X mutation in primary human bronchial epithelial (HBE) cells from CF patients with compound heterozygous mutations of R553X-F508del. They achieved an average correction rate of 83.7% for the desired product with only moderate bystander editing. In mouse models, LNP-ABE treatment was administered to heterozygous R553X mice carrying one allele with the humanized R553X mutation and one normal mouse *CFTR* allele. 10 days post-treatment, they observed a 50.0% correction at the targeted position in lung stem cells, 12.2% in the entire lung, and 28.7% in the trachea. This study demonstrated that lung-targeting SORT LNPs can effectively deliver BEs to lungs while genomic editing in lung stem cells presents a promising long-term therapeutic strategy for genetic lung diseases.

#### 4.3.4 LNPs for the treatment of DMD

Ionized cationic LNPs are popular vehicles for gene editing due to their ability to undergo charge reversal in neutral extracellular fluid before cellular uptake and ionization in low-pH endosomes for membrane fusion. However, encapsulating Cas9 RNPs into LNPs is challenging due to the need for acidic conditions, which can denature Cas9. Wei et al.^82^ addressed this by introducing permanently cationic lipids, such as 1,2-dioleoyl-3-trimethylammonium-propane (DOTAP), into LNPs. Through evaluation using a GFP reporter assay, these optimized LNPs demonstrated approximately 80.9% indels after 5 days of co-incubation with GFP-HeLa cell lines. By adjusting the molar percentages of DOTAP, the LNP complexes exhibited organ targeting from liver to lung, which is particularly useful for targeted gene editing in specific organs. In a DMD exon 44 deletion mouse model, immunofluorescence analysis confirmed that injection of RNPs-encapsulated LNPs into tibialis anterior muscles led to the restoration of dystrophin expression. The strategy was also effective across various LNP types, including stable nucleic acid lipid particles and lipid-like nanoparticles, for gene editing in brain and liver tissues. This study advances RNP packaging methodology and provides an effective strategy for organ-selective RNP delivery.

#### 4.3.5 LNPs for the treatment of SCD

Daniel et al.^174^ utilized bone-marrow (BM)-homing LNPs to edit hematopoietic stem cells, reactivating fetal hemoglobin and converting sickle to non-sickle alleles in a mouse model of SCD (*HBB*^*S/S*^ mice). To target cells within the BM niche, covalent-bond-forming lipid species were incorporated into the LNPs, enhancing surface enrichment of proteins like apolipoprotein E (ApoE) for BM delivery (Fig. 6B). This design improved transfection efficiency across various BM cell types, including HSCs, progenitor cells, B cells, T cells, macrophages, monocytes, neutrophils, and endothelial cells. CRISPR-Cas9 gene editing was utilized to disrupt the *BCL11A* binding motif and induce fetal hemoglobin production in *HBB*^*S/S*^ mice. After two intravenous injections of BM-homing LNPs containing *Cas9* mRNA and sgRNA, mutations were detected in HSPCs within 7 days. Indels were present in 5.2% of alleles within hematopoietic stem and progenitor cells (HSPCs). In another approach, ABE8e_NRCH was used to convert the sickle cell disease allele to the non-pathogenic Makassar allele. NGS revealed that 2.43% of the sickle cell disease alleles were converted to the Makassar allele by injecting BM-homing LNPs into mice. These findings suggest a novel therapeutic strategy for treating SCD and provide insights into the development of targeted treatments for genetic blood disorders.

#### 4.3.6 LNPs for the treatment of eye diseases

*In vivo* genome editing with RNPs is safer than using viral Cas9 systems, but transient RNP activity often results in suboptimal editing. In a more recent study, Hołubowicz et al.^175^ developed LNP formulations optimized for enhanced delivery of RNP complexes to treat inherited retinal degeneration caused by Rpe65 mutations in a mouse model (*rd12* mice). The efficiency of delivering RNPs was further enhanced by cell-penetrating peptides (CPP), either covalently fused to the protein or used as excipients, while the LNPs were optimized to improve RNP stability, delivery efficiency, and editing potency. Through optimization, the team achieved over a 300-fold increase in *in vivo* editing efficiency compared to the delivery of naked RNPs. These optimized LNPs encapsulated ABE and PE, demonstrating successful delivery and editing within the RPE. Following subretinal injections, precise genomic corrections were observed, with a notable restoration of visual function as evidenced by improvements in electrophysiological responses, including the pupillary light reflex and cortical activity.

#### 4.3.7 Current safety concerns and challenges of LNPs

LNP-based delivery enables precise, organ-targeted gene editing, offering a versatile platform for treating genetic diseases. However, challenges remain in their application. LNPs can stimulate innate immunity, which influences adaptive immune responses and may lead to diverse effects, such as autoimmune reactions and inflammation^212^. The existing LNP vaccines have shown systemic side effects such as pain, inflammation, and, in rare cases, myocarditis and/or pericarditis^213^. The lipid components, such as PEGylated lipids, may activate immune responses, causing inflammation, complement activation, and antibody production^214^. Additionally, cationic lipids can induce cell damage and inflammation through interactions with cellular membranes^215^. Commercial-scale production of LNPs faces further obstacles, including the need to meet strict regulatory standards and the difficulty of scaling up manufacturing processes. Addressing these issues requires continued innovation in LNP design to enhance their effectiveness and safety for mRNA delivery applications.

### 4.4 VLPs

Extracellular vesicles (EVs) are nanoscale particles released by cells and play a crucial role in intercellular communication during various physiological and pathological processes^216^^,^^217^. These versatile vesicles serve as carriers for diverse cellular cargo, encompassing a wide range of molecules, including proteins, nucleic acids, and soluble small molecules^217^. Furthermore, specific components within EVs possess inherent cargo transport capabilities that enable tissue-targeted delivery^216^. Given these intrinsic characteristics, researchers have been intrigued to explore the potential of EVs as a platform for drug delivery^218^.

**Figure 7:**
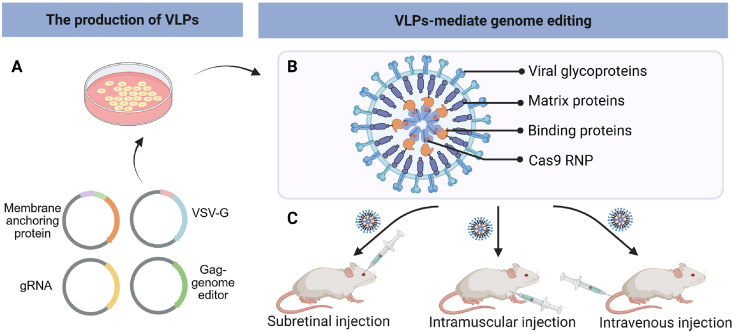
The production, structure, and application of VLPs. (A) Schematics of VLPs production from producer cells. (B) Schematics of VLPs. VLPs usually contain viral glycoproteins, matrix proteins, binding proteins, and genome editing proteins and/or RNA, such as Cas9 RNP. (C) VLPs have been utilized for delivering gene editing tools to eyes, muscles, and circulatory system to treat genetic diseases.

In addition to the natural release of EVs from parental cells, certain viral pathogens propagate virions into the extracellular environment using EV biogenesis mechanisms^219^. Research into viral biogenesis and assembly has paved the way for the development of VLPs. These particles are typically produced by expressing viral structural proteins in recombinant systems (Fig. 7A). VLPs composed of one or more viral scaffolds capable of self-assembly without containing viral genetics (Fig. 7B)^220^. By harnessing several key viral characteristics, VLPs offer efficient delivery systems, including targeting specific cell types, effective internalization in cells, and evasion of endosomal degradation, making them ideal for the temporary delivery of gene editing machinery for the treatment of genetic disorders^219^^,^^221^. Currently, VLPs have been used in preclinical trials to treat genetic diseases through subretinal injection, intramuscular injection, and intravenous injection (Fig. 7C).

#### 4.4.1 VLPs for the treatment of DMD

Gee et al.^176^ developed a novel EV delivery system, NanoMEDIC, for transporting spCas9 and sgRNAs to induce dystrophin exon 45 skipping in patient-derived induced pluripotent stem cells (iPSCs) with DMD. Exon 45 skipping has the potential to restore the reading frame in DMD patients lacking exon 44^222^. The researchers utilized an FKBP12 and FRB dimerization system to encapsulate the spCas9 protein, while selecting Gag as a candidate for packaging sgRNA. Additionally, they expressed vesicular stomatitis virus glycoprotein (VSV-G) on the surface of EVs to enhance broad tropism. The producer cells used for generating NanoMEDIC were 293T cells. By designing two sgRNAs targeting splicing acceptor and donor sites, NanoMEDIC achieved an impressive deletion of 92% of exon 45 in Δexon 44 DMD iPSCs. In luciferase reporter mice, the injection of NanoMEDIC into the gastrocnemius muscle consistently resulted in gene skipping for more than 160 days. In another study, Yao et al.^49^ used aptamers for selective enrichment of RNPs within EVs. They facilitated fusion between Cas9 and CD63, abundant in exosomes. Administration of EVs encapsulating RNPs targeting DMD exon 53 into Δexon 52 DMD mouse models resulted in an approximate induction of 0.2% indels in the tibialis anterior muscle.

#### 4.4.2 VLPs for the treatment of eye diseases

Age-related macular degeneration (AMD) is a prevalent cause of blindness worldwide, affecting approximately 20% of the elderly population. Wet AMD involves neovascularization-induced damage to the neural retina. Ling et al.^177^ investigated the therapeutic potential of VLP-mediated delivery for targeted suppression of vascular endothelial growth factor A gene (*Vegfa*) using the CRISPR-Cas9 system in the treatment of AMD. They created an all-in-one VLP vector by transfecting 293T cells with plasmids for *Cas9* mRNA and gRNA. Cas9 was fused with the MS2 stem-loop, and the Gag polyprotein (Gag-Pol) facilitated the packaging of the gRNA cassette. Following subretinal injection and a 14-day observation period, Ling et al.^177^ observed a reduction in VEGFA levels to approximately 10 pg/mL in the RPE-choroid-scleral (RCS) complex compared to approximately 40 pg/mL in the control group. Pre-treatment with VLP-CRISPR also prevented choroidal neovascularization, with no immune response detected in the treated mice.

In another investigation, Banskota et al.^178^ used VLPs to restore visual function in mouse models of LCA, a genetic disease characterized by retinal degeneration and blindness^178^^,^^223^. Mutations in the *RPE65* gene account for approximately 6% of all LCA cases, making it a promising target for *in vivo* gene editing interventions^223^. Banskota and colleagues^178^ developed engineered DNA-free VLPs (eVLPs) using the murine leukemia virus (MLV) Gag-Pol as scaffolds. ABE was tethered to the Gag-Pol *via* a linker peptide, which undergoes cleavage during particle maturation. Additionally, the researchers modified the eVLPs with nuclear export signals (NESs), along with conventional NLSs, to ensure cytoplasmic localization in the producer cells. In the LCA mouse model harboring a nonsense mutation in exon 3 of *Rpe65*, ABE-eVLPs facilitated a significant on-target A·T > G·C conversion rate of 21% in RPE tissue five weeks post-injection, surpassing the 11.5% correction achieved by lentivirus encoding ABEs. The system’s versatility was further demonstrated by its ability to correct mutations in the central nervous system and knock out *PCSK9* in the liver.

These studies highlight the potential of genetically engineered VLPs as efficient carriers for RNA-based CRISPR-Cas9 and ABE systems, offering promising therapeutic strategies for inherited eye diseases.

#### 4.4.3 VLP for treatment of HT1

Mangeot et al.^179^ presented two potential strategies for the treatment of HT1 utilizing a novel type of MLV-based VLPs named Nanoblades. By fusing spCas9 to the C-terminal end of the Gag protein and co-transfecting HEK-293T cells with plasmids containing all the necessary components, they successfully packaged the Cas9 RNP complex within the Nanoblades. Firstly, they efficiently edit the *Tyr* gene in embryos, with an efficiency of 16 out of 40. After embryo transplantation, 5 out of 8 F_0_ mice exhibited varying degrees of *Tyr* disruption, demonstrating the potential of Nanoblades for *in vivo* gene editing applications. Secondly, they investigated *in vivo* editing targeting the *HPD* gene. Through retro-orbital injection, Nanoblades achieved editing efficiencies ranging from 7% to 13% *in vivo*^224^. Overall, this study demonstrates an efficient delivery method for Cas9 and sgRNA using flexible and easily prepared VLPs.

#### 4.4.4 Comparison of VLPs to other non-viral vectors

VLPs are particularly attractive among non-viral systems because (i) they mimic the structure and function of viruses, enabling selective binding to target cells or tissues, which improves their delivery efficiency, and (ii) they naturally or through engineering (*e.g.*, RGD peptide insertion) facilitate precise, cell-specific delivery. Meanwhile, they still face challenges compared to other non-viral vectors (*e.g*., LNPs). First, VLPs often trigger stronger immune responses due to viral structural proteins. While this immunogenicity enables VLPs to induce both antibody-mediated and cell-mediated immune responses, making them a potent vaccine platform, it may also limit their suitability for repeated dosing^225^^,^^226^. In contrast, LNPs generally exhibit lower immunogenicity. Second, VLPs have limited cargo capacity, struggling to deliver large CRISPR/Cas9 systems as single constructs. The use of split-Cas9 dual-vector systems can bypass this limitation^177^, and recent advancements in VLPs have progressively addressed this issue^178^^,^^227^. LNPs better accommodate large nucleic acids like full-length *Cas9* mRNA, though encapsulation efficiency drops for oversized payloads. Third, VLP production is costlier and complex, relying on protein expression in mammalian/insect cells and precise assembly. LNPs, synthesized *via* scalable chemical methods (*e.g*., microfluidics), are more industrial-friendly.

In conclusion, VLPs excel in targeting accuracy and transfection efficiency, whereas LNPs prioritize modular design and scalability. In clinical trials for the treatment of genetic diseases, VLPs have not yet yielded positive results as LNPs have. However, VLPs have demonstrated convincing efficacy in Human papillomavirus vaccines. Thus, we still anticipate that VLPs will demonstrate promising potential in delivering genome editors for the treatment of genetic diseases in the future.

### 4.5 Delivery methods for treating specific genetic diseases

In the above examples, we have highlighted the potential of non-viral delivery methods in treating genetic diseases. We briefly summarize the key features, advantages, and limitations of various non-viral delivery platforms, such as LNPs, exosomes, and viral vectors (*e.g*., AAVs), in Table 4. Because of the unique features of these delivery systems, selecting the appropriate vector is crucial depending on the target organ for specific genetic disorders.

**Table 4:** Comparison of delivery methods for treating genetic diseases.

Feature	AAV	Inorganic vector	Polymeric vector	Lipid-based vector	Virus-like particle
Safety	Potential risks like immunogenicity and harmful transgene insertion	Generally biocompatible, but may accumulate in certain organs	Biocompatible and biodegradable	Potential immune response issues, but less severe than viral vectors	Immunogenicity can be an issue due to viral protein content
Cargo restriction	Limited capacity for large genetic materials	No limit	No limit	No limit	Limited cargo capacity, but advancements are helping improve this
Transfection efficiency	High efficiency	Generally lower than viral vectors, but improves with modification	Generally lower compared to viral vectors, but improves with modifications	Generally good, especially for mRNA delivery	Better than other non-viral systems, but lower than viral vectors
Targeting capability	Can be engineered to target specific tissues or cells	Can be modified to target specific tissues, though less precise than viral vectors	Efficient transfection of lungs and spleen; weaker hepatic tropism	Efficient transfection of hepatocytes; spleen and lungs transfection	Excellent targeting precision
Advantages	High transduction efficiency; cell/tissue tropism; long-term expression for diseases requires durable editing	Scalable fabrication; flexibility in loading different molecules	Biodegradable; self-assembly; easy surface modification with ligands	Great biocompatibility; ability to deliver larger payloads	Highly targeted delivery; suitable for temporary gene delivery
Limitations	Immunogenicity; harmful transgene insertion; limited cargo capacity	Accumulation in organs; non-biodegradable; potential toxicity issues	Slower elimination of high-molecular-weight variants; difficulty in scaling up due to polydispersity	Potential toxicity, especially in higher doses; unstable output and loading efficiency	Higher cost of production; limited cargo capacity; potential immune responses

Abbreviations: AAV, adeno-associated virus.

LNPs are particularly effective for genetic diseases affecting the liver, as they predominantly distribute to the liver through systemic administration. LNPs can deliver genome editors to the liver *via* the ApoE–LDLR pathway, which has shown substantial success in treating conditions like ATTR (NCT06128629) and HAE (NCT05120830). Furthermore, SORT LNPs, which are administered *via* intravenous injection, have enabled selective lung transfection by incorporating a fifth component into the conventional LNP system, improving mRNA delivery specifically to the lungs in CF and other pulmonary disorders^125^^,^^211^. LNPs also hold promise for treating genetic diseases in other organs. For example, SORT LNPs can target the spleen to treat related conditions. In the case of eye diseases, ocular delivery of Cas9 RNP using LNPs^228^ has shown potential for safer gene editing by transiently expressing nuclease activity. Although non-viral delivery systems encounter significant challenges in treating brain diseases due to the blood–brain barrier (BBB), preclinical studies have demonstrated the feasibility of LNPs for brain tissue editing^82^^,^^229^. This may be attributed to the high drug loading capacity of LNPs, which makes them promising even with relatively low uptake into brain tissue.

VLPs are another promising non-viral system, offering efficient transfection and targeted delivery due to their virus-like structure. The eye is easily accessible in clinical settings and is isolated from the systemic immune system, making it suitable for VLPs, which have certain immunogenicity. By engineering envelope proteins for specific targeting, VLPs can precisely deliver gene editing therapies directly to retinal cells, offering promising treatments for inherited retinal conditions. For example, recent studies highlight VLPs as efficient carriers for RNA-based CRISPR-Cas9^177^ and ABE^178^ systems in ocular disease treatment. For hepatic disease, VLPs have shown potential in correcting liver genes for downregulating PCSK9 levels and treating HT1^178^^,^^179^. Regarding brain diseases, research has demonstrated that incorporating CPPs provides a viable strategy for VLPs to cross the BBB^230^. In general, VLPs are suitable for treating diseases that can be addressed through localized delivery or targeted to specific cell types.

In addition to LNPs and VLPs, polymeric vectors are promising gene delivery systems due to their structural diversity. Their customizable features—including adjustable molecular weight, copolymerization potential, and stereoregularity—provide extensive flexibility for tailored gene therapy designs^231^. For systemic delivery strategies, specialized nanoparticle formulations are being developed for liver-targeted and lung-targeted treatments^231^^,^^232^. Localized delivery strategies using polymeric vectors have also demonstrated therapeutic efficacy: intratracheal injection of chitosan has been used to treat SP-B deficiency^119^, while intratracheal injection of polymeric nanoparticles has successfully edited RPE cells^163^. Furthermore, inorganic nanoparticles, exemplified by CRISPR-Gold technology, have shown potential in delivering the CRISPR-Cas9 system for neurological^153^ and muscular diseases^164^. However, caution is warranted regarding GNPs, as their non-biodegradable nature raises concerns about potential bioaccumulation.

## 5 Summary and future perspectives

Genetic disorders present significant challenges, with many still lacking approved treatments. These diseases often lead to severe, life-threatening symptoms early in life. While conventional treatments, like small-molecule drugs and enzyme replacement therapies, have been developed for several genetic disorders, they are not applicable to all genetic diseases. Most of these therapies only manage symptoms rather than addressing the underlying cause and often require ongoing, repetitive administration, placing a substantial burden on patients. Genome editing has the potential to correct disease-causing germline mutations, providing new treatment opportunities for genetic diseases. AAV vectors have been extensively investigated as carriers for delivering gene editing drugs^5^^,^^92^^,^^118^^,^^233^^,^^234^. However, AAVs face challenges such as limited packaging capacity, detrimental transgene integration, off-target effects, and high production costs^5^^,^^235^.

In recent years, non-viral vectors, especially LNPs, have emerged as promising alternatives due to their increased loading capacities and favorable safety profiles. Recent advancements have made these non-viral vectors more feasible for clinical applications. For example, NTLA-2002 utilized liver-targeting LNPs to deliver CRISPR-Cas9 for editing the *KLKB1* gene, successfully reducing total plasma kallikrein levels in HAE patients during a phase 1/2 clinical study (NCT05120830)^173^. Similarly, NTLA-2001 utilized LNP delivery to edit the TTR gene in the livers of ATTR amyloidosis patients, resulting in decreased serum TTR levels while demonstrating a good safety profile, which led to its progression into a phase 3 study (NCT06128629)^169^.

To advance the clinical application of non-viral vectors for treating genetic diseases, several key issues must be addressed, including off-target effects, gene editing efficiency and specificity, and potential immune responses. Future research can address these issues by improving gene editing tools and designing rational non-viral delivery vectors. Off-target genome editing occurs when CRISPR-induced DNA cleavage and repair take place at unintended genome locations, often resembling the intended editing target’s sequence. Additionally, undesired genomic editing outcomes can arise from diverse DNA repair mechanisms following on-target DNA cleavage in different cells. These events may lead to serious consequences^236^, ^237^, ^238^, ^239^. To mitigate these risks, several Cas9 variants have been developed to minimize off-target effects while maintaining efficiency^240^, ^241^, ^242^. In addition, some new technologies provide alternatives to traditional CRISPR-based genome editing methods. For example, Durrant et al.^243^ introduced a novel bridge RNA system enabling direct DNA recombination through a compact RNA-guided recombinase, thereby expanding the toolkit for treating genetic diseases involving large deletions. Other considerations include the immunogenicity of bacterial editing proteins, pre-existing antibodies against CRISPR components, and uncertainties regarding the long-term safety and stability of genome editing results. However, the immunogenicity issues can be managed through high-efficiency one-time treatments or alternative enzymes. Additionally, *ex vivo* editing therapies, like those for sickle cell disease, are less affected, as residual CRISPR components decay naturally in edited cells. These strategies, along with protocol optimization, help mitigate potential immune responses^244^.

To improve therapeutic outcomes, it is crucial to not only use advanced editing tools to minimize off-target effects and enhance editing efficiency but also to rationally design delivery vectors that target specific organs and improve transfection efficiency. Currently, non-viral delivery systems are primarily used for hepatic diseases and vaccines due to their liver accumulation. Therefore, overcoming the liver tropism of nanoparticles while targeting other organs becomes crucial^231^. For example, the SORT strategy of LNPs involves specific components called SORT molecules that modify the *in vivo* delivery profile of LNPs, enabling precise targeting of organs such as the lungs, spleen, and liver. In addition, incorporating nanomaterials responsive to disease-specific conditions could further enhance their accumulation at the target site. For example, Xu et al.^245^ employed a poly(disulfide) and a liver-specific promoter to establish a dual liver-specific Cas-mediated DNA or RNA editing system. In another study, Chen et al.^246^ designed a nanoCRISPR comprising cationic polymer-coated Au nanorods, heat-shock promoter-driven Cas9 plasmids, and heat-shock factors to precisely control gene editing by fine-tuning NIR radiation time. In addition to these bioresponsive systems, several comprehensive reviews also explore additional strategies^247^^,^^248^.

Transfection efficiency, especially strategies for endosomal escape, is another concern for the translation of non-viral delivery systems from bench to bedside. The benefits of biomimetic vectors offer new perspectives on gene editing tool delivery methods. As highlighted in this review, VLPs combine high infection efficiencies of viral vectors with transient features associated with mRNA, protein, and RNP delivery, potentially leading to efficient and safe genome editing^249^^,^^250^. In a recent study, Kreitz et al.^251^ developed an extracellular contractile injection system (eCISs), which utilizes a syringe-like mechanism for direct protein delivery, including CRISPR-Cas9, directly into target cells. By recognizing specific receptors on the cell surface with engineered tail fibers, eCISs inject payloads through sheath contraction, enhancing delivery efficiency by bypassing the endosomal pathway and allowing targeted delivery to specific cell types.

In summary, as shown by examples summarized in this review, non-viral delivery of genome editing tools has emerged as a robust alternative for the treatment of genetic diseases. The CRISPR-Cas technologies have undergone significant development and optimization, resulting in the creation of powerful gene-editing tools such as BEs, PEs, and RNA editing. Pairing these gene editing tools with efficient non-viral delivery systems, including inorganic vectors, polymeric vectors, lipid-based vectors, and VLPs, has enabled a range of gene editing applications to treat genetic diseases, from proof-of-concept studies in animal models to successful therapeutic outcomes in humans. Though immunogenicity and safety remain concerns of CRISPR-based therapies, we envision that the advancements in Cas9 variants and other genome-editing modifications offer solutions to mitigate these risks. Continued development of more efficient and specific delivery vectors, along with the refinement of gene editing methods, will accelerate the progress of non-viral gene editing therapies to realize their full potential in treating human genetic diseases.

## Author contributions

Yuan Ping and Xiaohong Chen conceptualized and supervised the work. Jiamin Yang and Yuxuan Chen wrote the draft. All authors contributed to the discussion and writing of this manuscript.

## Conflicts of interest

Yuan Ping is a scientific cofounder of Ruidax. The other authors declare no competing interests.
